# PPARG dysregulation as a potential molecular target in adrenal Cushing's syndrome

**DOI:** 10.3389/fendo.2023.1265794

**Published:** 2023-11-30

**Authors:** Sharmilee Vetrivel, Mariangela Tamburello, Andrea Oßwald, Ru Zhang, Ali Khan, Sara Jung, Jessica E. Baker, William E. Rainey, Elisabeth Nowak, Barbara Altieri, Mario Detomas, Deepika Watts, Tracy Ann Williams, Ben Wielockx, Felix Beuschlein, Martin Reincke, Silviu Sbiera, Anna Riester

**Affiliations:** ^1^ Department of Medicine IV, LMU University Hospital, LMU Munich, Munich, Germany; ^2^ Division of Endocrinology and Diabetes, Department of Internal Medicine I, University Hospital, University of Würzburg, Würzburg, Germany; ^3^ Section of Pharmacology, Department of Molecular and Translational Medicine, University of Brescia, Brescia, Italy; ^4^ Department of Molecular and Integrative Physiology, University of Michigan Medical School, Ann Arbor, MI, United States; ^5^ Institute of Clinical Chemistry and Laboratory Medicine, Technische Universität Dresden (TUD)/Universitätsklinikum Carl Gustav Carus Dresden (UKD), Dresden, Germany; ^6^ Department of Endocrinology, Diabetology and Clinical Nutrition, University Hospital Zurich (USZ) and University of Zurich (UZH), Zurich, Switzerland

**Keywords:** transcriptome, hypercortisolism, rosiglitazone, adrenocortical cell line, steroidome, cortisol, primary bilateral macronodular hype

## Abstract

**Background:**

We performed a transcriptomic analysis of adrenal signaling pathways in various forms of endogenous Cushing’s syndrome (CS) to define areas of dysregulated and druggable targets.

**Methodology:**

Next-generation sequencing was performed on adrenal samples of patients with primary bilateral macronodular adrenal hyperplasia (PBMAH, n=10) and control adrenal samples (n=8). The validation groups included cortisol-producing adenoma (CPA, n=9) and samples from patients undergoing bilateral adrenalectomy for Cushing’s disease (BADX-CD, n=8). *In vivo* findings were further characterized using three adrenocortical cell-lines (NCI-H295R, CU-ACC2, MUC1).

**Results:**

Pathway mapping based on significant expression patterns identified PPARG (peroxisome proliferator-activated receptor gamma) pathway as the top hit. Quantitative PCR (QPCR) confirmed that *PPARG* (l2fc<-1.5) and related genes – *FABP4* (l2fc<-5.5), *PLIN1* (l2fc<-4.1) and *ADIPOQ* (l2fc<-3.3) – were significantly downregulated (p<0.005) in PBMAH. Significant downregulation of *PPARG* was also found in BADX-CD (l2fc<-1.9, p<0.0001) and CPA (l2fc<-1.4, p<0.0001). *In vitro* studies demonstrated that the PPARG activator rosiglitazone resulted in decreased cell viability in MUC1 and NCI-H295R (p<0.0001). There was also a significant reduction in the production of aldosterone, cortisol, and cortisone in NCI-H295R and in Dihydrotestosterone (DHT) in MUC1 (p<0.05), respectively.

**Outcome:**

This therapeutic effect was independent of the actions of ACTH, postulating a promising application of *PPARG* activation in endogenous hypercortisolism.

## 1 Introduction

Enhanced adrenal cell proliferation clinically translates into a high incidence of adrenal incidentaloma occurring in 2–10% of the population worldwide (1). One third of these patients have mild autonomous cortisol secretion without typical Cushing stigmata but associated with an elevated cardiometabolic morbidity (2–4). Comparatively, endogenous cortisol excess resulting in Cushing’s syndrome (CS) has an incidence of 0.2–5.0 per million people per year (5). In the majority of patients with overt CS, endogenous hypercortisolism is due to adrenocorticotropic hormone (ACTH) secretion by corticotroph adenomas of the pituitary gland resulting in Cushing’s disease (CD) (6). In approximately 20% of cases cortisol is secreted autonomously by the adrenal cortex. Adrenal CS is mostly caused by unilateral cortisol-producing adenomas (CPA). Primary bilateral macronodular adrenocortical hyperplasia (PBMAH) represents another specific subtype of adrenal CS characterized morphologically by bilateral nodular enlargement with a grape-like appearance. PBMAH accounts for less than 2% of patients with endogenous CS (7). Genomic approaches led to the identification of germline *ARMC5* mutations in approximately 20-25% of PBMAH patients, and germline *KDM1A* mutations in 90% of patients who have food-dependent CS (8). *PRKACA* gene mutations are detected in approximately 40% of cortisol-producing adenomas (9).

Despite the advances in genomic analyses, the medical management of CS remains controversial. Uni- or bilateral adrenalectomy is the first line treatment in overt CS. Patients after bilateral adrenalectomy require lifetime steroid replacement with risk of life-threatening adrenal crises (10, 11). Medical therapy in CS consists of steroid synthesis inhibitors and glucocorticoid receptor antagonists (12, 13). However, long term control of hypercortisolism with the available drugs has been afflicted with various side effects on gastrointestinal, neural and hepatic systems (14).

In this study our aim was to define altered cell signaling pathways in CS through PBMAH transcriptome and identify new pharmacological targets using the following experimental approach:

(1) Untargeted transcriptome analysis of PBMAH samples of the discovery cohort.
(2) Pathway analysis and preliminary validation of the next generation sequencing (NGS) results using QPCR.
(3) Validation of the candidate pathway genes including Peroxisome proliferator-activated receptor gamma (*PPARG*) pathway in an independent validation cohort.
(4) *In vivo* analyses of the effect of ACTH on *Pparg* expression
(5) *In vitro* experiments to assess the therapeutic effect of PPARG pathway modulation.

The overarching aim of our study was to have a comprehensive transcriptomics-based discovery dataset from patient samples that allows *in vitro* confirmatory studies. For this purpose, the unique genetic mutations and the increasing prevalence/missed diagnosis of PBMAH (13) make it an intriguing subtype of CS for the discovery cohort analyses. Further, by focusing on PBMAH, we aimed to identify previously unexplored therapeutic mechanisms for the CS pathology.

## 2 Materials and methods

### 2.1 Sample collection and ethics approval

The patients were registered as part of ongoing registries and biobanks (European Network for the Study of Adrenal Tumor [ENS@T, www.ensat.org] and Excellence Network for Neuroendocrine Tumors [NeoExNet]). The study was approved by the Ethics Committees of the University of Munich and Würzburg. Written informed consent was obtained from all enrolled patients, and the experiments were performed according to relevant guidelines and protocols.

For NGS a total of 18 cryo-preserved adrenal samples were used. The discovery cohort consisted of PBMAH (n=10) and adjacent normal adrenal cortex from patients with pheochromocytoma (controls, n=8).

Additional QPCR validation was performed in RNA extracted from cryo-preserved tissue from the following samples: cortisol producing adenoma (CPA, n=9), and adrenal samples from patients undergoing bilateral adrenalectomy for persistent Cushing’s disease (BADX-CD, n=8). Normal adrenals from patients who underwent kidney surgery (normal adrenals, n=10), and adrenal samples from patients with aldosterone producing adenoma (APA, n=10) were used as controls for validation. Furthermore, the results were validated with data from 3’RNA sequencing of RNA extracted from FFPE tissues on an independent PBMAH cohort (PBMAH-FFPE, n=11).

The clinical characteristics of the groups are given in **Table 1**. In total, four PBMAH patients carried *ARMC5* mutations (2/10 PBMAH samples used in the discovery and 2/11 used in the validation cohort), and one patient from the validation cohort carried a *KDM1A* mutation.

**Table 1:** Clinical characteristics of the patient groups.

	N	Age at diagnosis [years]	Sex [%female]	baseline ACTH [pmol/L]	Cortisol 24h Urine [nmol/day]	midnight Cortisol [nmol/L]	Cortisol after 1 mg Dexamethasone [nmol/L]	methods	preservation of the tissue
Normal Range				0.9-13.4	138-414	<4.1	< 50		
Discovery cohort									
Controls (normal adrenals)	8	53 [48;58]	50	NA	NA	NA	NA	RNA Seq, QPCR	cryo
PBMAH	10	61 [59;68]	60	0.6 [0.4;0.7]	994 [619;1256]	15.6 [9.3;21.1]	250 [206;481]	RNA Seq, QPCR	cryo
Validation cohort									
CPA	7	52 [40;57]	62	0.4 [0.4;0.8]	1684 [1449;2120]	16.0 [12.7;24.3]	386 [326;431]	QPCR	cryo
BADX-CD	8	42 [37;52]	75	10.5 [7.8;12.1]	2211 [1949;2338]	21.1 [12.0;27.3]	373 [233;622]	QPCR	cryo
APA	10	47 [43;52]	70	NA	NA	NA	NA	QPCR	cryo
Normal adrenals	10	NA	NA	NA	NA	NA	NA	QPCR	cryo
PBMAH	11	58 [56;62]	100	0.6 [0.5;0.9]	458 [254; 530]	12.6 [8.2; 15.9]	155 [110;373]	3´ RNA Seq	FFPE

Data are given as median with 25th and 75th percentile in brackets. APA, Aldosterone producing adenoma; CPA, cortisol producing adenoma; BADX-CD, Bilateral adrenalectomized patients with persistent Cushing’s Disease. PBMAH, Primary Bilateral Macronodular Hyperplasia.

### 2.2 Total RNA extraction from cryo and FFPE tissues

The adrenal tissues were stored at -80°C. Total RNA isolation was carried out from all adrenal cortex samples using the RNeasy Tissue Kit (Qiagen, Germany). The isolated RNA was kept frozen at −80°C until further use. RNA yield and purity were measured using NanoDrop (Thermofisher Scientific, Germany). Quality control of the isolated RNA, RNA library preparation, and sequencing was performed by QIAGEN (Zymo Research, Irvine, US). In case of RNA from FFPE material, the tumor area was marked and 10 μm sections from five serial slides were collected under a stero microscope (Motic). Total RNA was extracted from the collected tumor using the AllPrep DNA/RNA FFPE kit (Qiagen) according to the manufacturer’s instructions. The RNA quantity and quality were determined using NanoDrop 2000 spectrophotometer (Thermo Fisher).

### 2.3 RNA sequencing

RNA-seq from frozen adrenal samples was performed at Qiagen, Hilden, Germany and sequencing from FFPE samples was done at the SysMed Core Facility, Würzburg. Briefly, RNA integrity and the absence of contaminating DNA were confirmed by Bioanalyzer RNA Nano (Agilent Technologies) and by Qubit DNA High sensitivity kits, respectively. QIAseq Stranded RNA Library Kits was used for library preparation. Sequencing was performed on Illumina NextSeq (single end read, 75 bp). Adapter and quality trimming were performed by the “Trim Reads” tool from CLC Genomics Workbench. Further, reads were trimmed based on quality scores. The QC reports were generated by the “QC for Sequencing Reads” tool from CLC Genomics Workbench. Read mapping and gene quantification were performed by the “RNA-seq Analysis” tool from CLC Genomics Workbench (15). In case of FFPE derived RNA, sequencing libraries were prepared using the QuantSeq 3’ mRNA-Seq protocol (Lexogen, Vienna, Austria), from 100 to 500 ng RNA. Single read sequencing (1 ×75bp) was performed on a NextSeq 550 platform (Illumina, San Diego, CA, USA). For RNA extracted from FFPE samples, sequencing libraries were constructed using the QuantSeq 3’ mRNA-Seq protocol from Lexogen (Vienna, Austria). The libraries were prepared using 100 to 500 ng of RNA. Subsequently, single-read sequencing with a read length of 75 bases (1 × 75bp) was carried out on a NextSeq 550 platform from Illumina (San Diego, CA, USA) (16).

### 2.4 Validation of RNA expression of related pathway genes

Differentially expressed RNAs identified through NGS were validated by QPCR. RNA concentration was assessed using a NanoDrop 2000 spectrophotometer (Thermo Fisher Scientific), and reverse transcription was performed on 50 ng of RNA using Superscript VILO reverse transcriptase (Thermo Fisher Scientific) according to the manufacturer’s instructions. The selection of a suitable housekeeping gene was carried out using the BestKeeper tool to determine the most stably expressed housekeeping gene (p-value <0.001) (15). In frozen adrenal samples of the discovery cohort, controls (n=8) and PBMAH (n=10), housekeeping genes *ACTB*, *GAPDH*, and *PPIA* were evaluated. For adrenocortical cell lines (NCI-H295R; n=10, CU-ACC2; n=10, and MUC1; n=10), the housekeeping genes *ACTB* and *PPIA* were assessed. *PPIA* was identified as the most stable reference gene for human adrenal samples, while *ACTB* was chosen as the reference gene for adrenocortical cell lines (**Figure S1**). QPCR was conducted using TaqMan Fast Universal PCR Master Mix (Thermo Fisher Scientific) on a Quantstudio 7 Flex Real-Time PCR System (Thermo Fisher Scientific), following the manufacturer’s TaqMan mRNA assay protocol. TaqMan probes used are listed in **Table S1**, and negative control reactions lacked cDNA templates. Each QPCR reaction used 5 ng of cDNA and was analyzed in technical triplicates. The PCR was carried out in a 20 μL mixture, with 10 μL of Master Mix, 1 μL of TaqMan probes, 5 μL of cDNA, and 4 μL of nuclease-free water. The qPCR was performed following the recommended thermocycling conditions on the Quantstudio 7 Flex Real-Time PCR System (Thermo Fisher Scientific) under the Fast mode, with an initial activation at 95°C for 20 sec and 40 cycles of 95°C for 1 sec and 60°C for 20 sec. Gene expression levels were quantified using the relative quantification method (17), normalized to the reference gene, to enable easier comparisons.

### 2.5 *In vivo* ACTH stimulation

ACTH stimulation tests were performed in 16 female mice (C57BL/6). Briefly, Jfemale mice representing each time point of injection). Briefly, 13-week-old mice were intraperitoneally injected with 1mg/kg of ACTH (Sigma Aldrich, Germany) and adrenals collected after 10, 30, and 60 min of injections (4 mice per time point). In addition, control adrenals were collected from mice at baseline conditions (0 min). Details of the experiment are published in (18). *Gapdh* was used as housekeeping gene in the QPCR. All mice were maintained in accordance with facility guidelines on animal welfare and approved by Landesdirektion Sachsen, Germany.

### 2.6 *In vitro* assays to evaluate pathway activation

The identified *PPARG* pathway was further characterized *in vitro* using three adrenocortical cell-lines (NCI-H295R, CU-ACC2 and MUC1), derived from patients with malignant adrenocortical carcinoma (ACC). The human NCI-H295R cell line, derived from a primitive ACC in a female patient, was obtained from the American Type Culture Collection (ATCC) and cultured as indicated by ATCC. MUC-1 cell line, established from a neck metastasis of an EDP-M-treated (etoposide, doxorubicin and cisplatin plus oral mitotane) male patient, was kindly given by Dr. Hantel and cultured as suggested (19). CU-ACC2 cell lines were obtained from Dr. Katja Kiseljak-Vassiliades (20). A detailed description of these cell lines can be found in Sigala et al. (21). The activation of *PPARG* in the cell lines was assayed for cell viability, gene expression changes and effect on steroidogenesis. Rosiglitazone, which is a known drug in diabetes therapy, was used as a *PPARG* activator.

### 2.7 Cell viability assay, hormones measurements and gene expression

Cells (50.000 cells/well) were seeded in 96-wells-plates and treated with increasing concentrations of ACTH (2.5–20 nM; Alfasigma), solubilized in water, and rosiglitazone (5–40 µM; Cayman Chemical). Rosiglitazone was solubilized and serially diluted using DMSO. The drug solutions were prepared 200 times more concentrated to dilute the DMSO at a ratio of 1:200 in the well. Both compounds were tested alone and in combination. Cell viability was evaluated by WST-1 assay according to the manufacturer protocol (Roche). All the subsequent sets of experiments were conducted treating the cells for 48h in serum-free medium. For treatments on CU-ACC2 cell line also the hydrocortisone has been removed.

Cells (1.5x10^6^ cells/well) were seeded in 6-wells-plates and treated with ACTH (2.5 nM), and 5, 10, 20 µM of rosiglitazone in a final volume of 3 ml. These doses were chosen based on the cell viability results. After 48h the media were collected and stored at -20°C until analysis performed using liquid chromatography tandem mass spectrometry (LC-MS/MS) as described by Schweitzer et al. (22). The cells were scraped from the bottom of the wells to isolate the mRNA using the Maxwell RSC Simply RNA Kit (Promega). RNA concentration was determined using a NanoDrop 2000 spectrophotometer (Thermo Fisher) and 1000 ng RNA were reverse transcribed with the High-Capacity cDNA Reverse Transcription Kit, Applied Biosystems). QPCR was performed using TaqMan gene expression probes (Thermo Fisher Scientific) for *PPARG* (Hs01115513) and for ACTH receptor, also known as the melanocortin receptor 2 (*MC2R*), (Hs00300820). Endogenously expressed *ACTB* (Hs99999903) was used as housekeeping gene for normalization. For each QPCR reaction, 5 ng cDNA were used, and each sample was analyzed in technical triplicates. All transcripts were amplified using TaqMan Gene Expression Master Mix (Thermo Fisher) using the CFX96 real-time thermocycler (Bio-rad) and the Bio-rad CFX Manager 2.0 software. Cycling conditions were 95°C for 3 min, followed by 39 cycles of 95°C for 30 s, 60°C for 30 s, and 72°C for 30 s. Fold change was calculated using the delta cycle threshold (dCt) method, normalized to housekeeping gene *ACTB*.

### 2.8 Bioinformatic and statistical analyses

R version 4.2.0 was used for statistical analyses of NGS data. To identify RNAs differentially expressed, generalized linear model (GLM, a flexible generalization of ordinary linear regression that allows for variables that have distribution patterns other than a normal distribution) in the software package edgeR (Empirical Analysis of DGE in R) was employed to calculate *p*-values (23, 24). *P*-values were adjusted with the Benjamini–Hochberg false discovery rate (FDR) procedure (25). GraphPad Prism Version 8 was used for statistical analysis of QPCR. To identify RNAs differentially expressed in QPCR, the dCt method (target gene’s Ct minus housekeeping RNA’s Ct) was used in Microsoft Excel 2016 (Microsoft, Redmond, WA, USA). For intergroup comparison of QPCR data, ANOVA test with Benjamini–Hochberg false discovery rate (FDR) procedure was used (26). P<0.05 and FDR<0.05 were considered significant. Pathway mapping for the significant genes was performed using the ShinyGO program (27).

## 3 Results

### 3.1 PBMAH transcriptome

**Figure 1:**
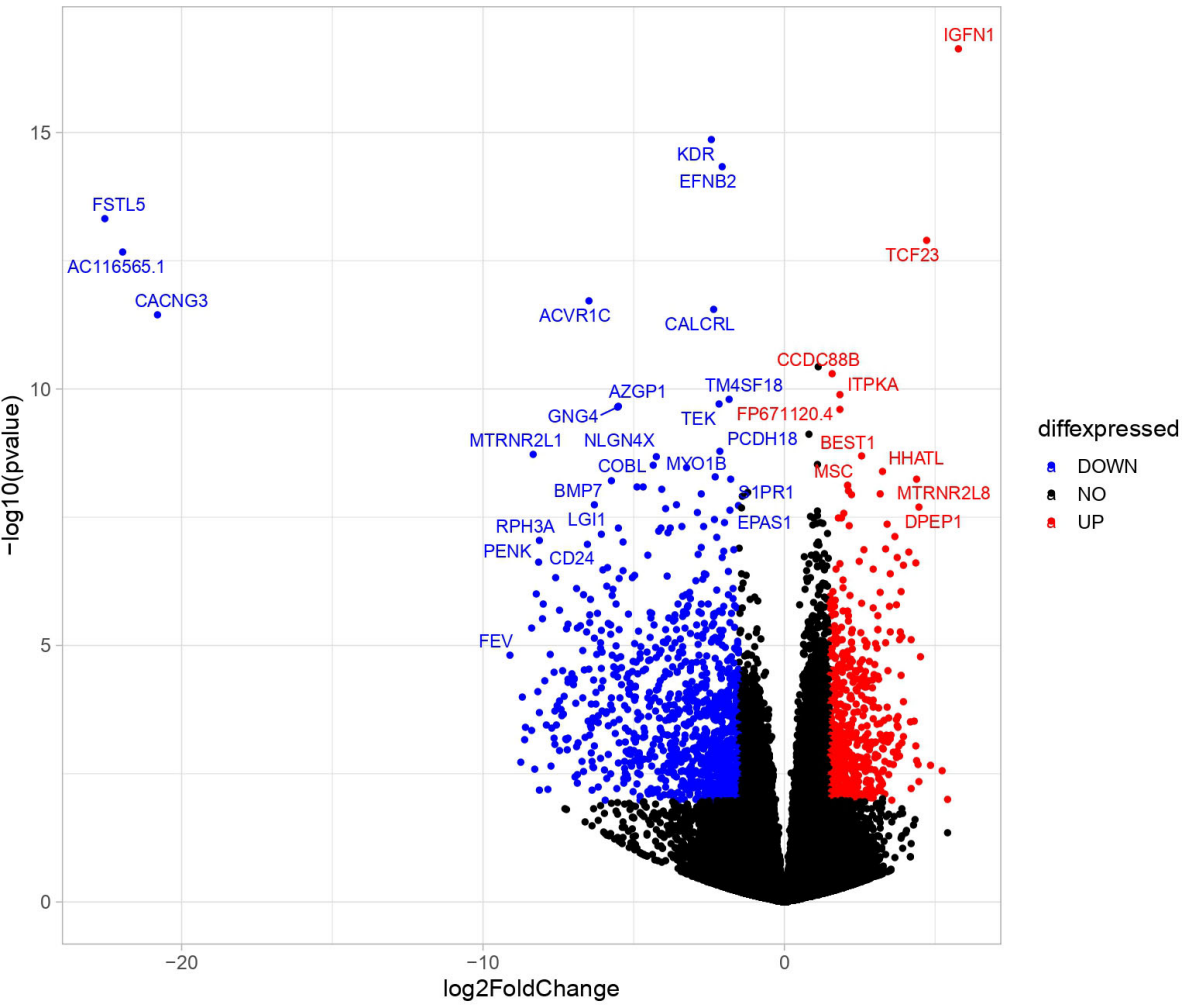
Differentially expressed significant genes in NGS between in adrenals of PBMAH vs. controls. Volcano plot showing the relationship between fold change (log2foldchange) and statistical significance (-log10pvalue). The red points represent significantly upregulated genes while blue points represent significantly downregulated genes. The top 32 altered genes are labelled (24 downregulated and 10 upregulated).

Transcriptome analyses of PBMAH adrenal samples (n=10) identified a total of 1104 genes to be significantly differentially expressed in comparison to the control group (n=8) in the discovery cohort (l2fc>|2|, FDR<0.05). Almost 70% of the genes were downregulated (769 genes), indicating an overall transcriptionally repressed state in PBMAH samples (**Figure 1**, **Table S2**). Hierarchical clustering based on the top upregulated significant genes was performed and revealed a good discrimination between (27)en PBMAH samples and controls. Interestingly, PBMAH samples with *ARMC5* mutations clustered together while the *ARMC5^wt^* PBMAH samples clustered separately and were further divided in two additional clusters (**Figure S2**).

### 3.2 Pathway mapping

**Figure 2:**
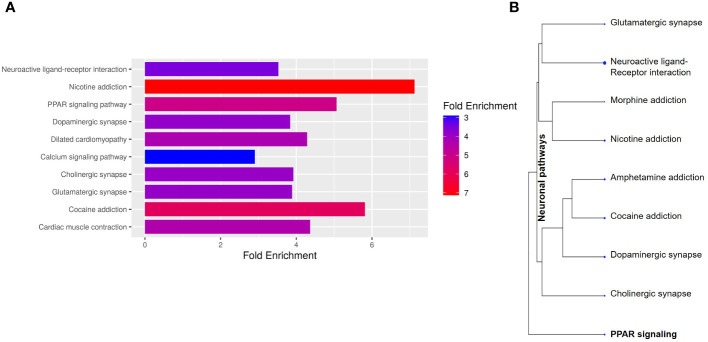
Pathway analyses of the significant genes from NGS. The significantly expressed genes were shortlisted (log2FC > abs (2), p<0.05). **(A)** The shortlisted genes were used for KEGG pathway mapping using ShinyGO online analyses tool. **(A)** Barplot representation of the top significant pathway hits. Higher fold enrichment refers to higher representation of the genes in the pathway. The pathways are sorted by FDR, with higher significant pathways at the top. **(B)** Hierarchical clustering of significant pathways: The pathway genes were clustered based on the genes shared amongst the different pathways.

**Figure 3:**
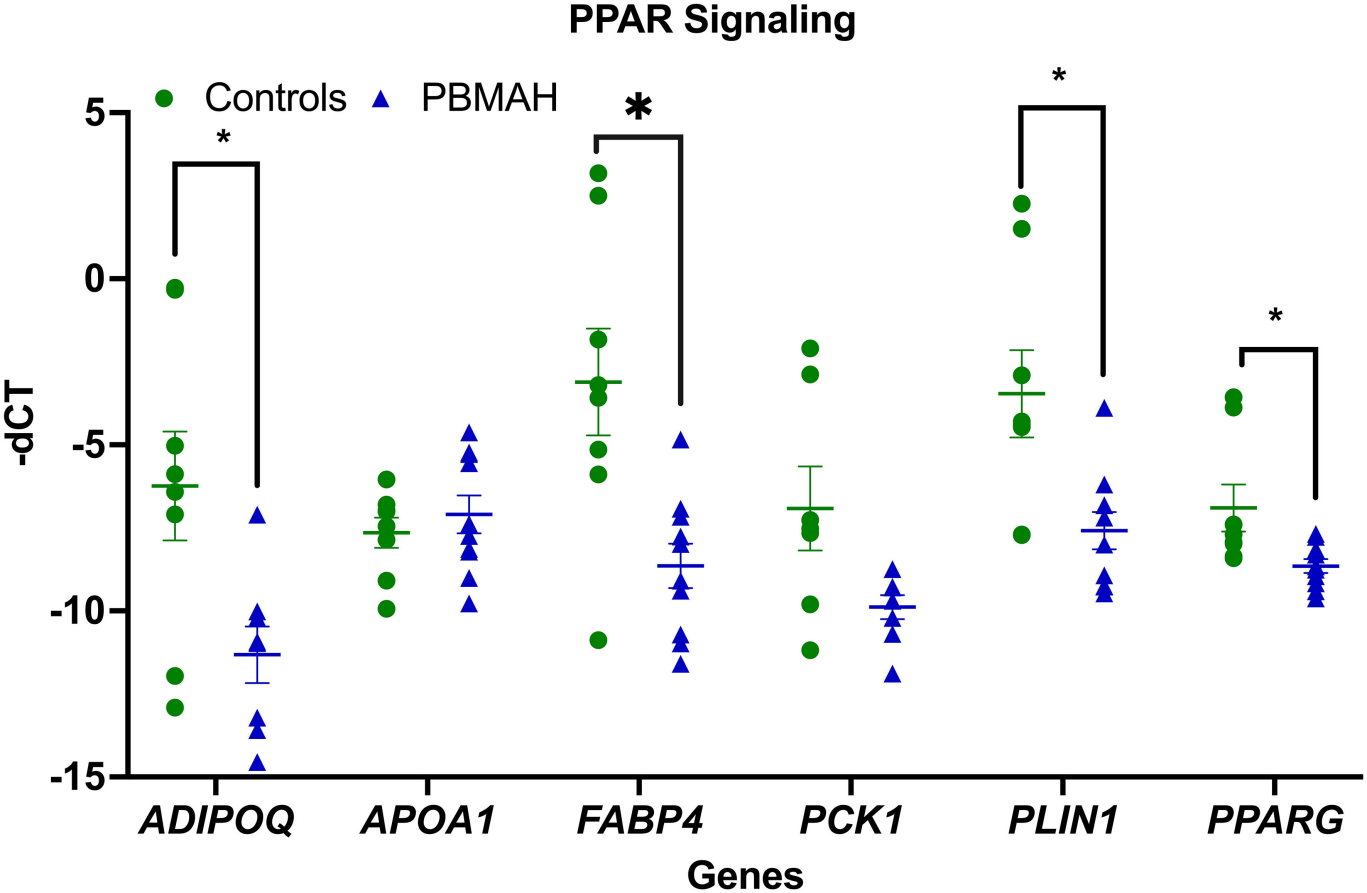
QPCR analyses of the significant genes from pathway analyses. Expression analysis of the pathway genes from PPARG signaling. Data are represented as mean ± standard error of mean (SEM) of -dCT values. Housekeeping gene: *Ppia*. *p-value <0.05 and FDR<0.05. PBMAH, Primary Bilateral Macronodular Hyperplasia.

To better understand the transcriptional profile of PBMAH, all significant genes (n=1104 genes) were used for pathway mapping to identify which genes are enriched in different pathways (**Figure 2A**). This pathway mapping analysis is grounded in the concept of fold enrichment (**Figure 2A**), wherein fold enrichment is computed as the percentage of genes in the input list relative to the corresponding background percentage. This calculation serves as a metric of the statistical significance of pathway dysregulation. Overall, the top pathway hits from the PBMAH transcriptome generated two major pathway clusters (**Figure 2B**) (1): “peroxisome proliferator-activated receptors” (PPARs) signaling, which is widely acknowledged for its anti-glycemic and anti-lipolytic effects (28), and (2) pathways related to neuronal function and diseases including “nicotine addiction” and “neuroactive ligand-receptor interaction”. To validate the pathway enrichment based on the transcriptome data, the significantly altered *PPARG* and its top downregulated target genes (*ADIPOQ*, *APOA1*, *FABP4*, *PCK1* and *PLIN1*) were chosen. A significant (p<0.01) downregulation of *PPARG* and its target genes *ADIPOQ*, *FABP4* and *PLIN1* was found in the PBMAH samples in comparison to controls (**Figure 3**). For the neuronal pathway cluster, five genes (*DRD2*, *GRIA2*, *GRIA4*, *GRIN2A* and *SCTR*) shared most among the pathways were chosen for further analyses but failed to show significant dysregulation (**Figure S3**).

### 3.3 Validation of PPARG pathway dysregulation

**Figure 4:**
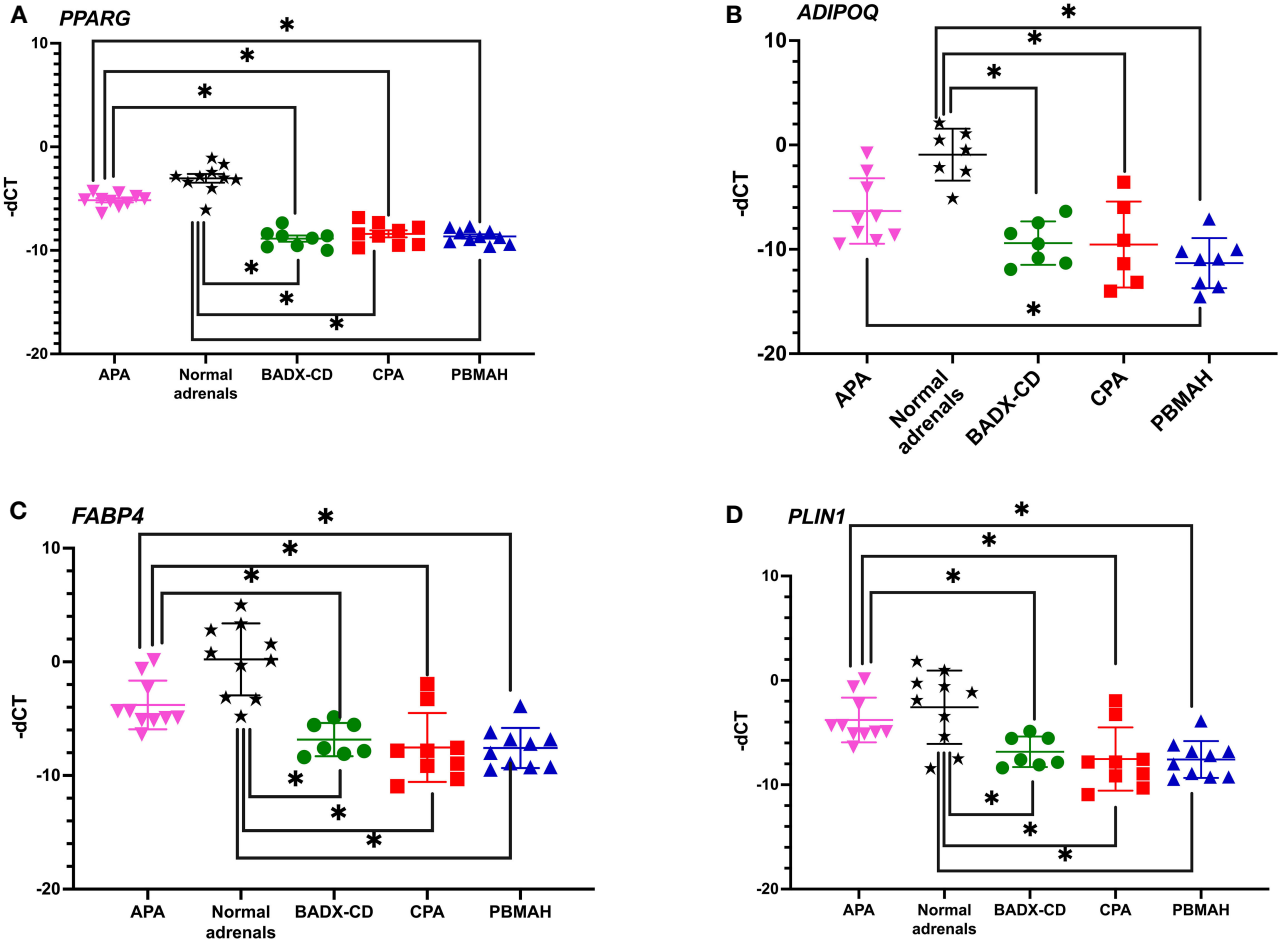
QPCR analyses of the significantly altered PPARG pathway genes in CS subtypes. The expression of significantly altered *PPARG* **(A)** and its target genes *ADIPOQ* **(B)**, *FABP4* **(C)**, *PLIN1* **(D)** were tested in CS subtypes of CD and CPA including additional controls of APA and normal adrenals. Data are represented as mean ± SEM of -dCT values. Housekeeping gene: *Ppia*. *p-value <0.05 and FDR<0.05. APA, Aldosterone producing adenoma; CPA, cortisol producing adenoma; BADX-CD, Bilateral adrenalectomized patients with persistent Cushing’s Disease.

The observed downregulation of *PPARG* and its target genes by QPCR and NGS were further validated in two ways. In the first step, DESeq2 based gene expression analyses from FFPE RNA from an independent PBMAH cohort (n=11) (**Table 2**) showed that *PPARG* and its target genes were consistently downregulated in the validation cohort of PBMAH. In the second step, the expression of *PPARG* and its target genes was verified in adrenal samples from other types of CS, normal adrenals and APAs (**Figure 4**). Significant downregulation of *PPARG* was observed in all the CS samples in comparison to both controls, APAs and normal adrenals (**Figure 4A**). Interestingly, significant downregulation of *PPARG* and its target genes, *ADIPOQ* (**Figure 4B**) and *FABP4* (**Figure 4C**) was also observed in APA in comparison to normal adrenals, but not as pronounced as in CS subtypes. In contrast, the significant downregulation of *PLIN1* was specific to CS groups (**Figure 4D**). Taken together, the candidate PPARG pathway downregulation observed in the adrenal samples of PBMAH by NGS could be validated in all adrenals with cortisol excess.

**Table 2:** Comparison of significant log2Fold Change (p<0.05) of PPARG and its target genes in the validation and discovery cohort of PBMAH in comparison to controls.

PBMAH vs Controls	Discovery Cohort (PBMAH)		Validation Cohort (PBMAH-FFPE)
Gene	NGS	QPCR	NGS
*ADIPOQ*	-5.8	-5.0	-8.4
*FABP4*	-2.3	-5.5	-6.1
*PLIN1*	-2.9	-4.1	-9.1
*PPARG*	-2.0	-1.7	-7.3

### 3.4 *In vivo* analyses of *Pparg*

**Figure 5:**
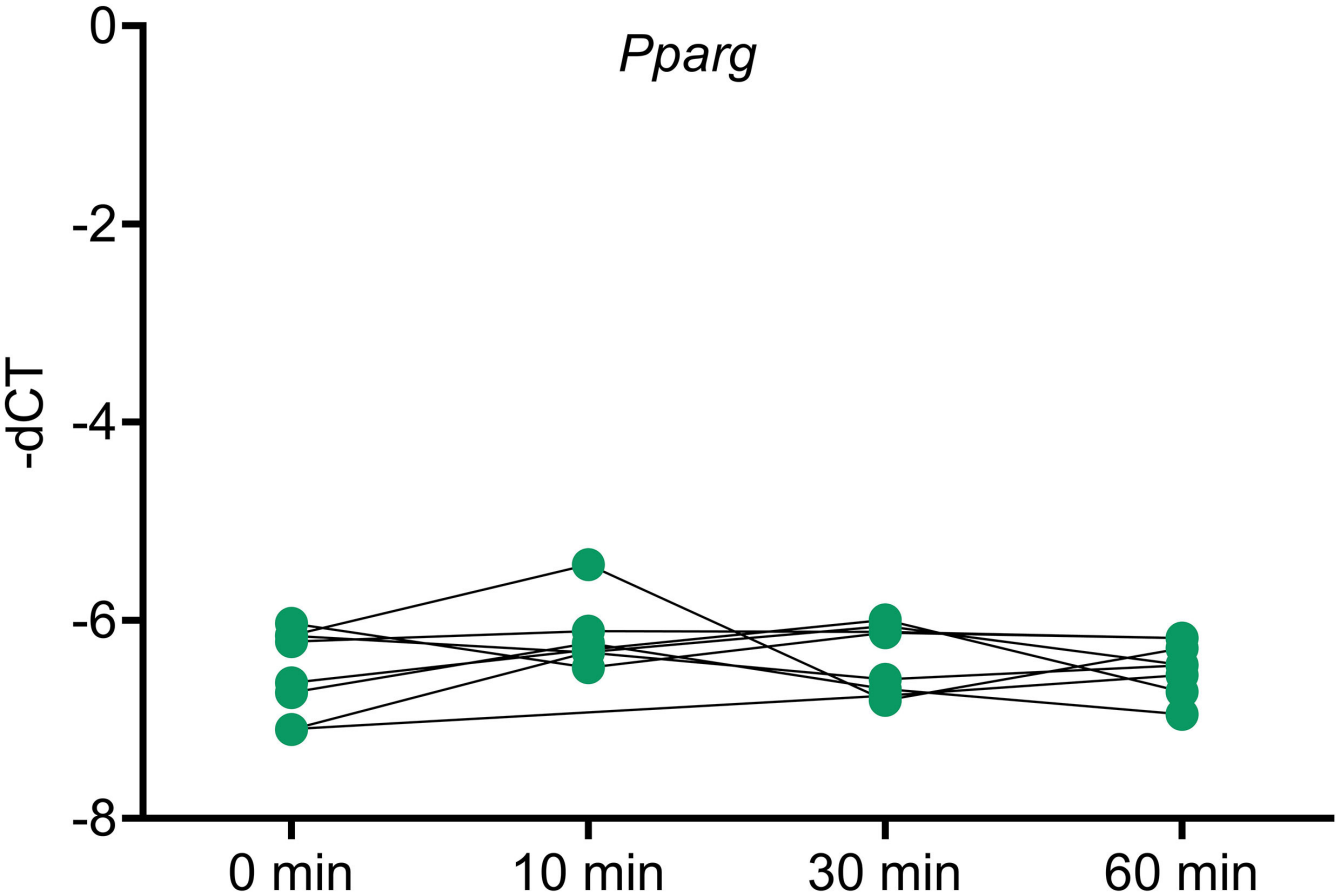
*Pparg* expression in ACTH stimulated murine adrenals. Mice were injected with ACTH and adrenals were collected at different timepoints after ACTH stimulation to assess the impact of ACTH on *Pparg* expression. Housekeeping gene: *Gapdh*. * <0.05 and FDR<0.05 (*).

To analyze whether *Pparg* expression is influenced by ACTH, an ACTH stimulation study was done in mice and *Pparg* expression was assayed at different timepoints. No changes in *Pparg* expression were found in any of the timepoints (**Figure 5**).

### 3.5 *In vitro* analysis of the PPARG dependent pathway activation

**Figure 6:**
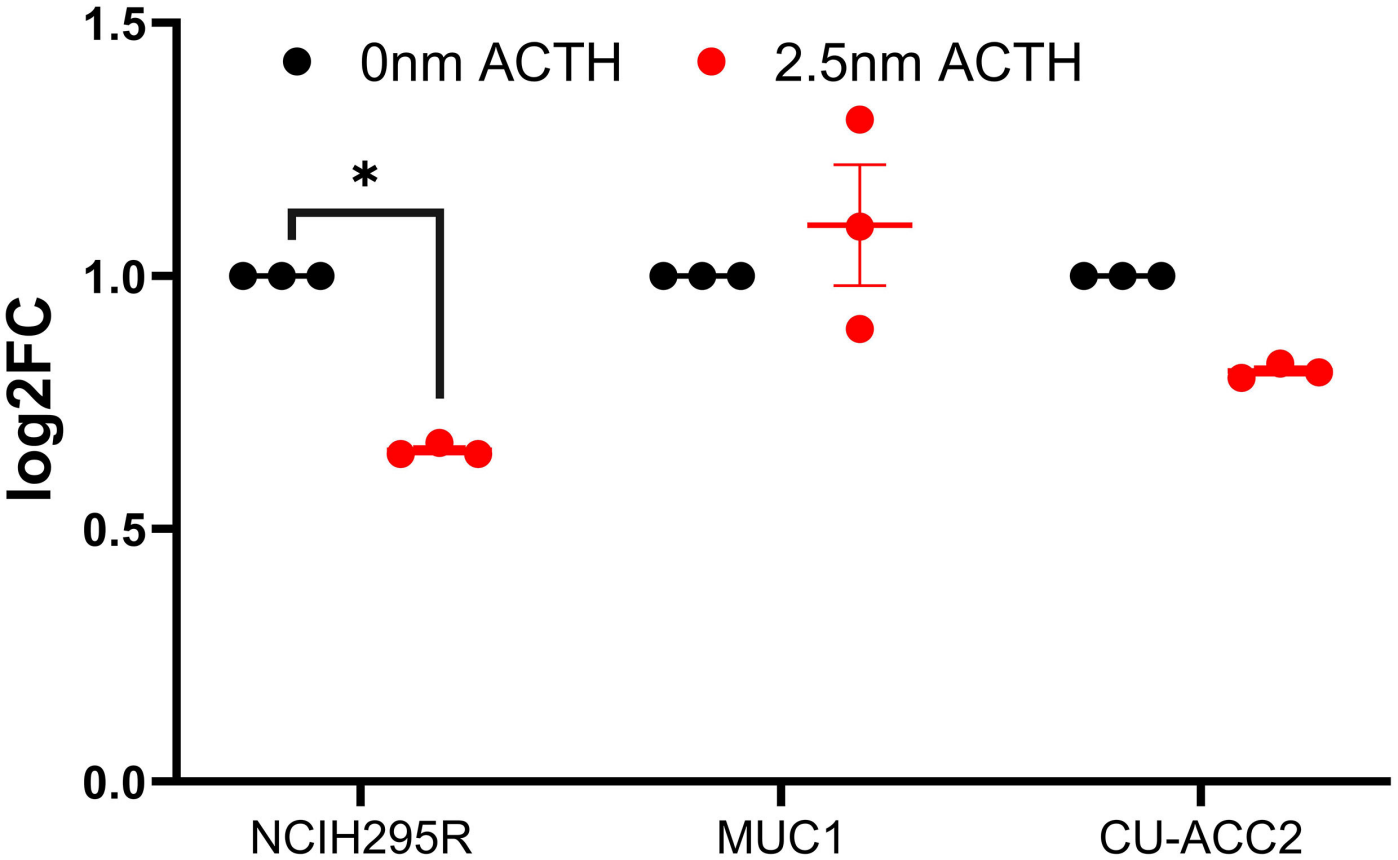
Effect of ACTH treatment on *MC2R* expression in adrenocortical cell lines. Data are represented as mean ± SEM of log2Fold Change (log2FC) expression values normalized to the control samples. Housekeeping gene: *ACTB*. *p-value <0.05 and FDR<0.01.

Next, the effect of ACTH stimulation on *MC2R* mRNA expression was tested in adrenocortical cell lines. Only NCI-H295R cells were found to show a decrease in *MC2R* expression upon ACTH treatment (**Figure 6**). The activation of PPARG in the cell lines was assayed for cell viability, gene expression changes, and effect on steroidogenesis. Rosiglitazone, an insulin sensitizer formerly used in diabetes therapy, was used as a PPARG activator.

#### 3.5.1 Cell viability

**Figure 7:**
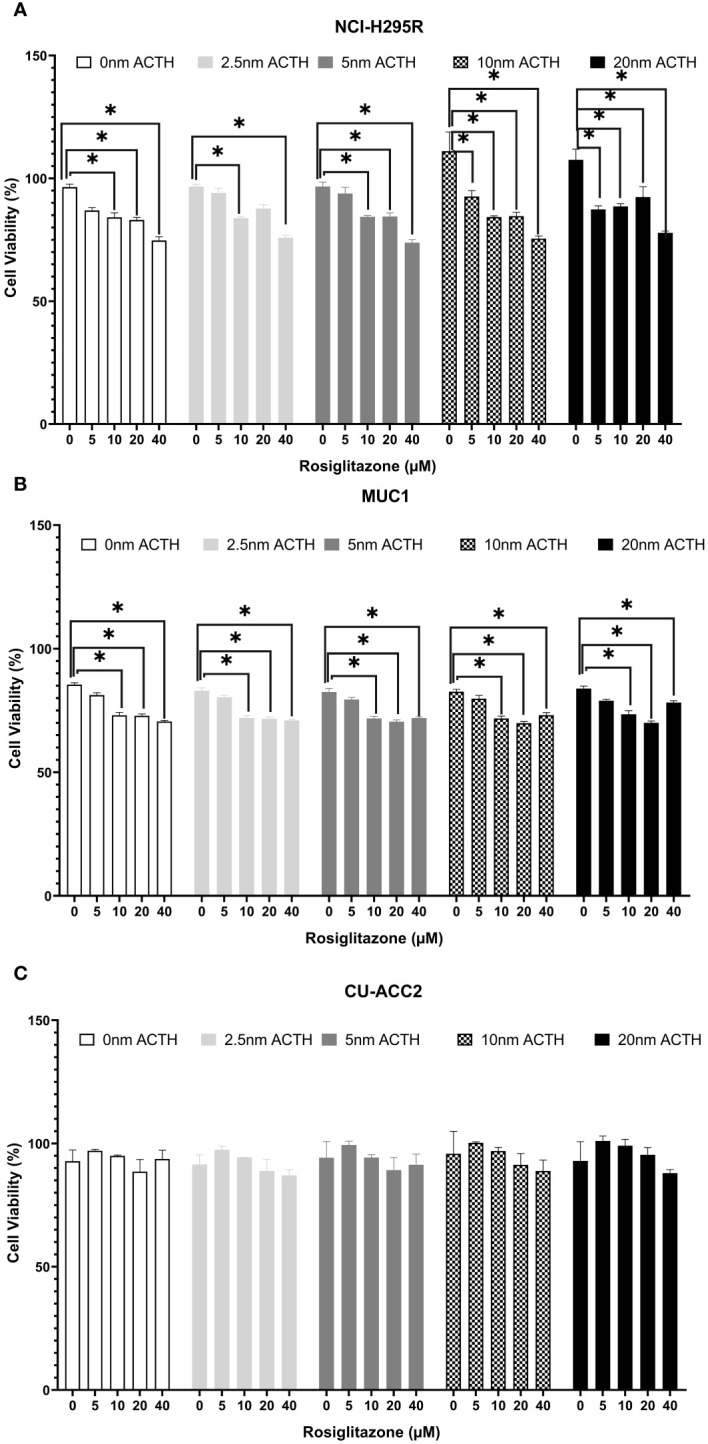
Effect of rosiglitazone and ACTH treatment on cell viability in three different adrenocortical cell lines: NCI-H295R **(A)**, MUC1 **(B)** and CU-ACC2 **(C)**. Cell viability was evaluated by WST-1 assay (n=8 in each group). The cells were treated with increasing concentrations of rosiglitazone (0, 5, 10, 20 and 40 µM) and ACTH (0, 2.5,5,10 and 20 nm). The average absorbance values of each treatment group were normalized to background control group. Data are represented as percentage mean ± SEM of the normalized absorbance values. *p-value <0.0005 and FDR<0.01.

Cell viability was assessed under varying ACTH and rosiglitazone concentrations. Rosiglitazone impaired NCI-H295R and MUC1 cell viability (**Figures 7A, B**): a trend of decreased cell viability was found using 5µM with significant reductions at the consecutive doses of 10, 20 and 40µM. ACTH stimulation did not interfere with the apoptotic effect of rosiglitazone as significant reduction in cell viability was found over all ACTH concentrations. In CU-ACC2 cells, rosiglitazone did not have any effect on cell viability in presence or absence of ACTH (**Figure 7C**).

#### 3.5.2 PPARG activation

**Figure 8:**
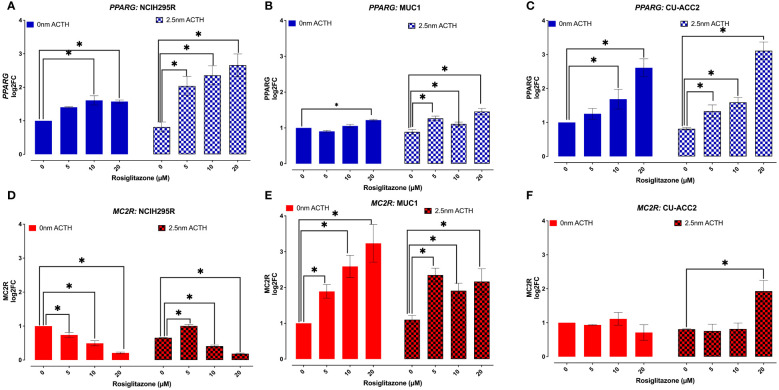
Effect of rosiglitazone and ACTH treatment on *PPARG* and *MC2R* expression in adrenocortical cell lines. The cells were treated with increasing concentrations of rosiglitazone (0,5,10,20 and 40 µM) and the expression was checked in the presence (2.5nm) and absence of ACTH. Data are represented as mean ± SEM of log2Fold Change (log2FC) expression values normalized to the control samples. Housekeeping gene: *ACTB*. *p-value <0.05 (*).

*PPARG* and *MC2R* expression were assessed in the absence or presence of ACTH (2.5nM), to understand whether the observed loss in cell viability was a direct effect of *PPARG* activation (**Figure 8**). In the absence of ACTH, all tested cell lines showed *PPARG* expression to increase with increasing doses of rosiglitazone. Briefly, significant increased *PPARG* expression was found at 10 and 20µM of rosiglitazone in NCI-H295R (**Figure 8A**), at 20µM of rosiglitazone in MUC1 (**Figure 8B**), and at 10 and 20µM of rosiglitazone in CU-ACC2 (**Figure 8C**). In the presence of ACTH, a more pronounced *PPARG* activation was found with significantly increased *PPARG* expression at all analyzed doses (**Figures 8A–C**). In case of *MC2R*, the cell lines showed varying results: In NCI-H295R, rosiglitazone treatment led to significantly downregulated *MC2R* expression, both in presence and absence of ACTH (**Figure 8D**). MUC1 cells showed upregulated *MC2R* expression both in the presence and absence of ACTH (**Figure 8E**). In CU-ACC2 cells, no changes in *MC2R* expression were found in the absence of ACTH. However, in the presence of ACTH, rosiglitazone at 20µM was found to significantly increase *MC2R* expression (**Figure 8F**).

#### 3.5.3 Steroidome

**Figure 9:**
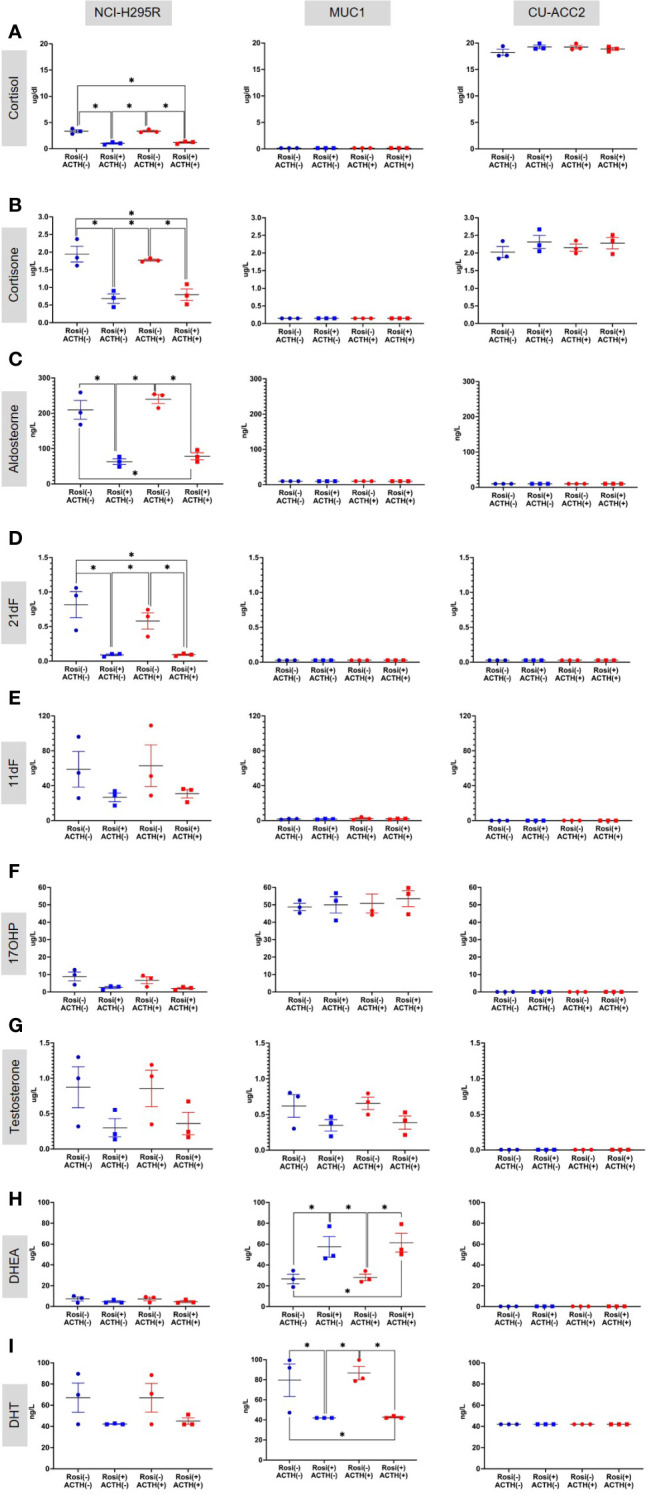
Effect of rosiglitazone (20µM) and ACTH (2.5 nm) treatment on the steroidome of adrenocortical cell lines. LC-MS/MS was used to quantify the steroidome in the supernatant of cells treated with rosiglitazone and ACTH and their respective controls. Importantly, the levels of glucocorticoids – cortisol **(A)**, cortisone **(B)** and aldosterone **(C)**, precursors of cortisol – 21-deoxycortisol [21-dF;**(D)**], 11-deoxycortisol [11-dF; **(E)**] and 17-hydroxyprogesterone [17OHP;**(F)**], androgens – testosterone **(G)**, DHEA **(H)** and dihydrotestosterone [DHT;**(I)**] were quantified. Data are represented as mean ± SEM of individual concentration values (µg/L). *p-value <0.05 and FDR<0.05 (*).

Based on these findings, the rosiglitazone treatment of 20µM was chosen to estimate the effect of rosiglitazone on steroidogenesis in all cell lines. Briefly, LC-MS/MS analyses revealed that rosiglitazone treatment led to a 30% reduction in cortisol levels in NCI-H295R cells, from a mean value of 3.32 ± 0.42 µg/dl to 1.00 ± 0.19 µg/dl(**Figure 9A**). The reduction was maintained in the presence of ACTH stimulation with a reduction from 3.24 ± 0.24 µg/dl to 1.181 ± 0.19 µg/dl. Rosiglitazone was also found to significantly reduce cortisone (**Figure 9B**), aldosterone (**Figure 9C**), and 21-deoxycortisol (21-df; (**Figure 9D**) levels in NCI-H295R. In NCI-H295R, no other significant changes were observed (**Figures 9E–I**, **S4**, **S5**). In case of MUC1, no detectable levels of cortisol, cortisone, aldosterone, 21-deoxycortisol and 11-deoxycortisol were seen (**Figures 9A–E**). However, a significant increase in DHEA levels coupled with a significant reduction in DHT levels was found upon rosiglitazone treatment (**Figures 9H, I**). The significant change was also maintained in the presence of ACTH in MUC1. There was no change in the other androgens and precursor levels (**Figure S5**), including testosterone (**Figure 9G**). Interestingly, CU-ACC2 cells had higher cortisol and cortisone levels compared to both MUC1 and NCI-H295R, but without changes upon rosiglitazone and/or ACTH treatment (**Figures 9A, B**). The observation was confirmed to be a property of the CU-ACC2 cells and not affected by the hydrocortisone in the media by repeating the experiment with medium containing no hydrocortisone (data not shown).

#### 3.5.4 PPARG target genes

**Figure 10:**
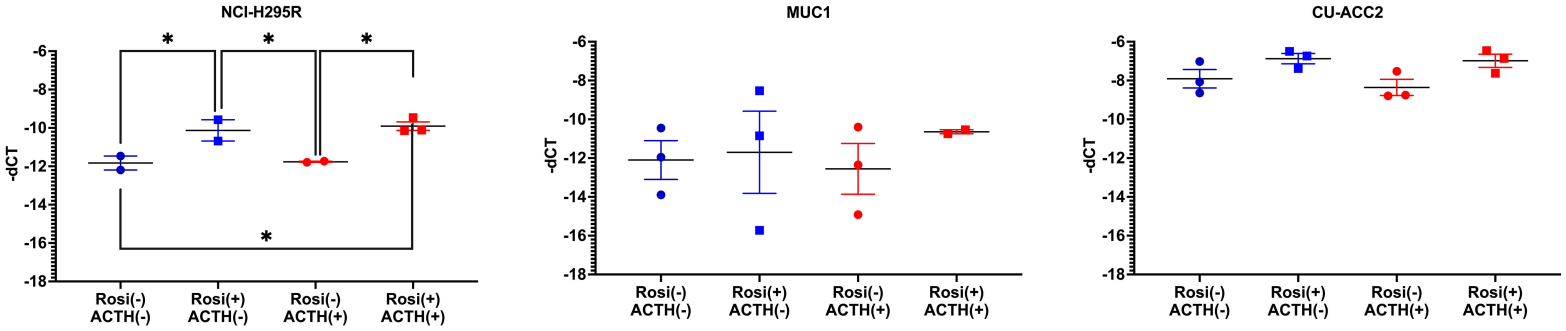
Expression of *PLIN1*, PPARG target gene, in adrenocortical cell lines treated with rosiglitazone (20µM) and ACTH (2.5 nm). Data are represented as mean ± SEM of -dCT values. Housekeeping gene: *ACTB*. *represents p-value <0.05 and FDR<0.05 (*).

Finally, the expression of PPARG target genes was analyzed at 20µM rosiglitazone concentration in comparison to the controls (0µM rosiglitazone), in presence and absence of ACTH (2.5nmACTH). Interestingly, no expression of *ADIPOQ* was found in any of the cell lines (no amplification by QPCR, data not shown). Expression of *FABP4* was observed only in NCI-H295R and in CU-ACC2 on a low level but with no major changes upon rosiglitazone treatment (**Figure S6**). In case of *PLIN1*, expression was observed in all the cell lines, however, significant activation by rosiglitazone was observed only in NCI-H295R (**Figure 10**).

## 4 Discussion

Previous molecular approaches on the pathology of PBMAH have been directed primarily towards molecular heterogeneity (29) and genetic predispositions involving *ARMC5* (30) and *KDM1A* (11). Herein, we present a transcriptomic profile of PBMAH in comparison to controls. This enabled a targeted characterization of the molecular factors that are dysregulated in genes attest to this point. The highly different transcriptome profile with more than 1000 dysregulated genes attest to this point. Interestingly, the top dysregulated genes included *IGFN1* (l2fc=5.7, pvalue<0.005) and *TCF23* (l2fc=5.2, p-value<0.005) that had been previously identified by Di Dalmazi, Altieri et al. (31) in RNAseq analyses of CPA. In the same study neuronal pathways were found to be differentially expressed in mild autonomous producing adrenocortical adenomas and ACC, which is in line with our findings. Taken together, it could be speculated that neuronal pathway genes could represent a major differentially regulated pathway group in adrenal pathology. Validation of the pathway by QPCR identified a trend towards downregulation but it was not found to be significant.

Interestingly, the druggable pathway of PPARG signaling was identified as another top hit. *PPARG* downregulation is known to occur in pituitary corticotropic cells of CD patients (32), however, this is the first identification of its dysregulation in adrenals of CS patients. The pathway dysregulation was validated in the PBMAH samples of the discovery cohort as well as in additional cohorts of tumors associated with hypercortisolism such as CPA and BADX-CD. Also, in adrenal samples of APA, downregulation of *PPARG* and its target genes were found. This is in line with previous findings from Williams et al. (33). However, downregulation of *PPARG* pathway and its target genes were much more pronounced in the adrenal samples of CPA, BADX-CD and PBMAH in comparison to APA.

Since the first clinical application of PPARG agonists, thiazolidinediones (TZD) in 1997, there have been various studies exploiting the activation of PPARG pathway in various pathologies, including CD, regulation of steroidogenesis and adrenocortical tumors with varying results (34). Therefore, a comprehensive analysis of PPARG activation was done in three different adrenocortical cell lines – NCI-H295R, MUC1 and CU-ACC2 – varying concentrations of ACTH and rosiglitazone. These adrenocortical cell lines were chosen for experimentation due to the absence of established benign *in vitro* models for CS. Interestingly, none of the cell lines used in our study were to show upregulated *MC2R* expression, characteristic of adrenocortical cells to increase steroidogenesis via This MC2R mediated activation initiates a signaling cascade of. 3′,5′-cyclic AMP, protein kinase A, hormone-sensitive lipase, and steroidogenic acute regulatory protein (10). Rather, the NCIH295R cells showed a downregulation of *MC2R* upon ACTH stimulation (**Figure 6**) with no accompanying changes in steroidogenesis (**Figure 10**). Interestingly, downregulated *MC2R* expression have been observed in cortisol secreting adrenocortical carcinomas (35–37) and studies in canine models of ACC have suggested that cortisol regulates interrenal expression of *MC2R* in response to ACTH in a negative short-loop feedback (36, 38, 39). It could be speculated that the downregulated *MC2R* expression we found in the NCIH295R cells could be an indication of this negative short-loop feedback and with respect to this reaction in presence of ACTH, the NCIH295R cells could be said to be ACTH responsive. However, it should be noted that further mechanistic elaboration of the response is beyond the scope of the current study. In NCI-H295R cells rosiglitazone treatment decreased cell viability (**Figure 7A**) and transcriptionally activated *PPARG* at all ACTH concentrations (**Figure 8A**). The *PPARG* activation was also found to modulate *MC2R* expression, leading to its downregulation, both in the presence and absence of ACTH (**Figure 8A**). It could be hypothesized that in NCI-H295R, rosiglitazone reduces the *MC2R* expression to counteract the effects of ACTH mediated cortisol production. Furthermore, among the PPARG target genes only *PLIN1* was found to show upregulated expression upon rosiglitazone treatment in the cell line (**Figure 10**). Similarly, the downregulation of *PLIN1* was found to be specific only to CS, in contrast to *ADIPOQ* and *FABP4*, which were also downregulated significantly in APA (**Figure 4**). Interestingly, PLIN1 has been characterized to have a critical role in lipolysis (40) and has been associated with cholesteryl ester droplets in steroidogenic adrenocortical cells (41). In summary, PPARG activation by rosiglitazone in NCI-H295R cells alleviates hypercortisolism by cell death, via *MC2R* and the target gene of *PLIN1*. Considering the complex interplay among *PPARG*, *MC2R*, and *PLIN1*, it could be hypothesized that the modulation of PPARG by rosiglitazone could reduce suppressed lipid-mediated signaling. This could be particularly relevant in conditions characterized by suppressed inflammatory responses, such as CS and adrenocortical cancer.

In comparison to the promising effects in NCI-H295R, MUC1 and CU-ACC2 cells showed varying results in response to rosiglitazone treatment, where we observed a loss in cell viability only in MUC1 (**Figure 7B**) accompanied by an upregulation of *MC2R* and *PPARG* expression (**Figures 8C, D**). Interestingly, in one of the earlier studies performed on the primary NCI cell strains, rosiglitazone was also found to increase *MC2R* expression, decrease cell viability and promote steroidogenesis (42). Steroidome analyses showed that MUC1 cells have altered levels of DHT and DHEA upon rosiglitazone treatment. The effect of rosiglitazone treatment on DHT, DHEA levels in MUC1 combined with altered aldosterone and cortisone levels in NCI-H295R hint at an extended therapeutic effect of rosiglitazone on a range of adrenocortical steroids including cortisol. Contrarily, CU-ACC2 cells secrete cortisol but did not show any significant cell death or reduction in cortisol levels in response to rosiglitazone treatment (**Figure 7C**). Of note, CU-ACC2 cells had generally higher levels of cortisol and cortisone as NCI-H295R, however, no detectable level of their precursors (**Figure 9**). Therefore, this discrepancy in the modulation effects induced by rosiglitazone in the steroidogenically active NCI-H295R and CU-ACC2 could be the result of a suppressed classical cortisol synthesis pathway in CU-ACC2. The variability in cortisol secretion among the cell lines could also be explained by the varied malignant genetic background of the cell lines (43). Therefore, the consistently observed *PPARG* and *MC2R* regulation by rosiglitazone in the adrenocortical cell lines holds potential for prospective CS therapies.

In summary, we found the PPARG pathway to be prominently downregulated in adrenals in different CS subtypes including CPA and CD. The dysregulation was observed in all samples, irrespective of the mutational status, indicating that it is strongly linked to hypercortisolism and not to the specific genetic backgrounds found in CS. The *in vitro* activation using rosiglitazone yielded promising results with significant effects on cell viability, gene expression and steroidogenesis depending on the adrenocortical carcinoma cells used (**Table 3**). Therefore, rosiglitazone therapy has the potential of providing a therapeutic for a subset of adrenal CS patients as well, including PBMAH. This aligns with the findings from clinical trials in Cushing’s disease (CD), where rosiglitazone demonstrated the ability to reduce cortisol levels in a subgroup of patients (44, 45). Nevertheless, it is imperative to emphasize that additional research is essential to validate this potential fully. Given that Cushing is a metabolic disorder, our established *in vitro* models may not encompass all the necessary elements to comprehensively investigate the therapeutic effects of PPARG pathway. Therefore, the incorporation of primary cultures derived from CS patients could offer a valuable alternative for future investigations. Moreover, a targeted therapy designed for ectopic receptors in PBMAH (46), can be combined with rosiglitazone to potentially augment treatment outcomes. Furthermore, conducting mechanistic studies to elucidate the interplay between the PPARG pathway and pathological processes like inflammation and cortisol synthesis would provide a more comprehensive understanding of rosiglitazone’s application as a therapy for CS.

**Table 3:** Cumulative analyses of the effect of rosiglitazone treatment on adrenocortical cell lines.

Effects of Rosiglitazone treatment on	NCI-H295R	MUC1	ACC2
Cell viability	Reduced	Reduced	No change
ACTH responsiveness	Responsive	No change	No change
*PPARG* expression	Activated	Activated	Activated
*MC2R* expression	Downregulated	Upregulated	Upregulated (2.5nm ACTH)
Steroidogenesis	aldosterone, cortisol and cortisone levels decreased	No cortisol production	dihydrotestosterone (DHT) levels decreases
Target genes	Perilipin-1 activated	No change/low expression	No change/low expression

## Data availability statement

Table of the significant genes from the NGS data are deposited Ain the supplementary data (**Table S2**). As Cushing, being a rare disease, the sequence information provided could be traced back to the patients and therefore, not readily available because of data protection. However, reasonable requests (for research purpose) to access the complete datasets should be directed to the corresponding author.

## Ethics statement

The study was conducted according to the guidelines of the Declaration of Helsinki and approved by the Ethics Committee of the Ludwig Maximilian University, Munich (protocol code 379-10, 152-10 and 20 July 2021). Informed consent was obtained from all subjects involved in the study. The studies were conducted in accordance with the local legislation and institutional requirements. The participants provided their written informed consent to participate in this study. All mice were maintained in accordance with facility guidelines on animal welfare and approved by Landesdirektion Sachsen, Germany. The studies were conducted in accordance with the local legislation and institutional requirements. Written informed consent was obtained from the owners for the participation of their animals in this study. Written informed consent was obtained from the individual(s) for the publication of any potentially identifiable images or data included in this article.

## Author contributions

SV: Formal Analysis, Methodology, Software, Supervision, Writing – original draft, Conceptualization, Visualization, Data curation, Investigation. MT: Investigation, Methodology, Validation, Writing – review & editing. AO: Writing – review & editing. RZ: Writing – review & editing. AK: Methodology, Writing – review & editing. SJ: Methodology, Writing – review & editing. JB: Methodology, Writing – review & editing. WR: Validation, Writing – review & editing. EN: Investigation, Writing – review & editing. BA: Investigation, Writing – review & editing. MD: Investigation, Writing – review & editing. DW: Investigation, Writing – review & editing. TW: Investigation, Resources, Writing – review & editing. BW: Resources, Writing – review & editing. FB: Writing – review & editing. MR: Funding acquisition, Resources, Writing – review & editing. SS: Conceptualization, Funding acquisition, Resources, Validation, Writing – original draft, Writing – review & editing. AR: Conceptualization, Data curation, Funding acquisition, Project administration, Resources, Supervision, Validation, Writing – original draft, Writing – review & editing.
