# Supplementary material for: PPARG dysregulation as a potential molecular target in adrenal Cushing's syndrome
Source: Front Endocrinol (Lausanne). 2023 Nov 30;14:1265794. doi: 10.3389/fendo.2023.1265794 (PMC10720662; doi:10.3389/fendo.2023.1265794)
Supplement: Supplementary file 1 [file DataSheet_1.zip › supplementary 2023/Supplementary legends.docx]

**FigureS1:** BestKeeper analyses for the housekeeping genes in frozen adrenal samples (A;n=28) and in adrenocortical cell lines (B;n=30). Data is represented as Cp (Crossing point) values of the samples plotted against the respective samples. BestKeeper computes the pair-wise correlation analysis of the housekeeping genes. The coefficient of correlation (r) and the p-value of the genes are given. Genes that

**Figure S2:** Heatmap of the PBMAH transcriptome in comparison to the controls.

The top significantly expressed genes between PBMAH and controls were used for the hierarchical clustering analyses between the sequencing samples. The genotype of the PBMAH samples based on *Armc5* mutation status is also given.

**Figure S3:**  QPCR analyses of the significant genes from neuronal signalling pathway analyses.

Expression analysis of the neuronal pathway genes. Data are represented as mean ± standard error of mean (SEM) of -dCT values. Housekeeping gene: Ppia. * p-value <0.05 and FDR<0.05. PBMAH, Primary Bilateral Macronodular Hyperplasia.

**Figure S4:**  Effect of rosiglitazone (20µM) and ACTH (2.5 nm) treatment on aldosterone precursors in adrenocortical cell lines.

The precursors of aldosterone – Corticosterone (A), Deoxycorticosterone (DOC; B) and Progesterone (C) – were detected in the supernatant of cells treated with rosiglitazone and ACTH and their respective controls by LC-MS/MS analyses. Data are represented as mean ± SEM of individual concentration values (µg/L).

**Figure S5:**  Effect of rosiglitazone (20µM) and ACTH (2.5 nm) treatment on andorgens in adrenocortical cell lines.

The andorgens – A4; Androstenedione (A), DHEAS (B) and Estradiol (C) – were detected in the supernatant of cells treated with rosiglitazone and ACTH and their respective controls by LC-MS/MS analyses. Data are represented as mean ± SEM of individual concentration values.

**Figure S6:**  Expression of *Fabp4*, PPARG target gene in adrenocortical cell lines treated with rosiglitazone (20µM) and ACTH (2.5 nm).

Data are represented as mean ± SEM of -dCT values. Housekeeping gene: *Actb*. * p-value <0.05 and FDR<0.05 (*).
