# Supplementary material for: PPARG dysregulation as a potential molecular target in adrenal Cushing's syndrome
Source: Front Endocrinol (Lausanne). 2023 Nov 30;14:1265794. doi: 10.3389/fendo.2023.1265794 (PMC10720662; doi:10.3389/fendo.2023.1265794)
Supplement: Supplementary file 1 [file DataSheet_1.zip › supplementary 2023/Table S1.docx]

Table S1: List of genes and the related Taqman probes used in QPCR.

| Gene | Taqman Probe |
| --- | --- |
| *ACTB* | Hs99999903 |
| *ADIPOQ* | Hs00605917_m1 |
| *APOA1* | Hs00163641_m1 |
| *DRD2* | Hs00241436_m1 |
| *FABP4* | Hs01086177_m1 |
| *GRIA2* | Hs00181331_m1 |
| *GRIA4* | Hs00898778_m1 |
| *GRIN2A* | Hs00168219_m1 |
| *MC2R* | Hs00300820 |
| *PCK1* | Hs00159918_m1 |
| *PLN1* | Hs00160173_m1 |
| *PPARG* | Hs01115513_m1 |
| *PPIA* | Hs04194521_s1 |
| *SCTR* | Hs01085380_m1 |
| *ACTB* | Hs99999903 |
| *Pparg*_mouse | Mm00440940_m1 |
| *Gapdh*_mouse | Mm99999915_g1 |
