# Supplementary material for: PPARG dysregulation as a potential molecular target in adrenal Cushing's syndrome
Source: Front Endocrinol (Lausanne). 2023 Nov 30;14:1265794. doi: 10.3389/fendo.2023.1265794 (PMC10720662; doi:10.3389/fendo.2023.1265794)
Supplement: Supplementary file 1 [file DataSheet_1.zip › supplementary 2023/Table S1.pdf]

Table S1: List of genes and the related Taqman probes used in QPCR.

Gene	Taqman Probe
ACTB	Hs99999903
ADIPOQ	Hs00605917_m1
APOA1	Hs00163641_m1
DRD2	Hs00241436_m1
FABP4	Hs01086177_m1
GRIA2	Hs00181331_m1
GRIA4	Hs00898778_m1
GRIN2A	Hs00168219_m1
MC2R	Hs00300820
PCK1	Hs00159918_m1
PLN1	Hs00160173_m1
PPARG	Hs01115513_m1
PPIA	Hs04194521_s1
SCTR	Hs01085380_m1
ACTB	Hs99999903
*Pparg*_mouse	Mm00440940_m1
*Gapdh*_mouse	Mm99999915_g1
