# Supplementary material for: PPARG dysregulation as a potential molecular target in adrenal Cushing's syndrome
Source: Front Endocrinol (Lausanne). 2023 Nov 30;14:1265794. doi: 10.3389/fendo.2023.1265794 (PMC10720662; doi:10.3389/fendo.2023.1265794)
Supplement: Supplementary file 1 [file DataSheet_1.zip › supplementary 2023/Table S2.pdf]

Table S2: List of the differentially regulated genes in PBMAH transcriptome in comparison to the controls.

Gene	log2FoldChange	padj	sig
FSTL5	-22.000	0.0000	FDR<0.05
CACNG3	-21.564	0.0000	FDR<0.05
CNTNAP4	-21.440	0.0000	FDR<0.05
AC116565.1	-21.381	0.0000	FDR<0.05
AL445524.2	-20.836	0.0000	FDR<0.05
TBC1D3G	-16.707	0.0004	FDR<0.05
AC005943.1	-9.165	0.0009	FDR<0.05
FEV	-9.120	0.0057	FDR<0.05
TNR	-8.760	0.0081	FDR<0.05
SLC24A2	-8.689	0.0068	FDR<0.05
ADIPOQ	-8.669	0.0001	FDR<0.05
DRGX	-8.630	0.0077	FDR<0.05
GABRG2	-8.605	0.0046	FDR<0.05
PENK	-8.581	0.0005	FDR<0.05
UNC5D	-8.396	0.0155	FDR<0.05
MTRNR2L1	-8.332	0.0000	FDR<0.05
NEUROD4	-8.291	0.0167	FDR<0.05
GALR1	-8.228	0.0001	FDR<0.05
ST8SIA3	-8.189	0.0076	FDR<0.05
PHOX2B	-8.143	0.0104	FDR<0.05
TMEM271	-8.143	0.0336	FDR<0.05
ADGRA1	-8.040	0.0022	FDR<0.05
SYT2	-7.999	0.0097	FDR<0.05
ASPDH	-7.985	0.0052	FDR<0.05
RPH3A	-7.954	0.0009	FDR<0.05
NRSN1	-7.905	0.0098	FDR<0.05
PCSK2	-7.781	0.0036	FDR<0.05
CELF3	-7.746	0.0182	FDR<0.05
KCNQ2	-7.733	0.0070	FDR<0.05
ISL1	-7.639	0.0131	FDR<0.05
HAND1	-7.626	0.0104	FDR<0.05
MIR7-3HG	-7.611	0.0057	FDR<0.05
DBH	-7.607	0.0001	FDR<0.05
RD3	-7.591	0.0143	FDR<0.05
GDA	-7.576	0.0078	FDR<0.05
CHGA	-7.551	0.0067	FDR<0.05
NNAT	-7.472	0.0013	FDR<0.05
INSM1	-7.461	0.0219	FDR<0.05
SLC18A1	-7.456	0.0049	FDR<0.05
SST	-7.422	0.0038	FDR<0.05

SCRT1	-7.356	0.0031	FDR<0.05
GLB1L3	-7.351	0.0036	FDR<0.05
SPOCK3	-7.303	0.0101	FDR<0.05
AC087457.1	-7.253	0.0236	FDR<0.05
PHOX2A	-7.239	0.0091	FDR<0.05
SCG2	-7.234	0.0009	FDR<0.05
PCK1	-7.233	0.0010	FDR<0.05
TFAP2B	-7.222	0.0217	FDR<0.05
GRIA4	-7.201	0.0080	FDR<0.05
CPLX2	-7.178	0.0015	FDR<0.05
SULT4A1	-7.175	0.0029	FDR<0.05
SYT4	-7.172	0.0137	FDR<0.05
SCEL	-7.134	0.0130	FDR<0.05
RTL1	-7.055	0.0017	FDR<0.05
KSR2	-7.001	0.0078	FDR<0.05
DPYSL5	-6.988	0.0010	FDR<0.05
AC027228.2	-6.979	0.0056	FDR<0.05
AMER3	-6.952	0.0491	FDR<0.05
SLC35D3	-6.924	0.0023	FDR<0.05
KCNMB2	-6.907	0.0268	FDR<0.05
CHD5	-6.898	0.0008	FDR<0.05
DGKK	-6.880	0.0409	FDR<0.05
COL22A1	-6.878	0.0003	FDR<0.05
LINC00682	-6.857	0.0169	FDR<0.05
CADPS	-6.779	0.0011	FDR<0.05
CHRM1	-6.712	0.0385	FDR<0.05
TH	-6.708	0.0060	FDR<0.05
SLC18A2	-6.698	0.0020	FDR<0.05
MIR137HG	-6.678	0.0313	FDR<0.05
ARHGAP36	-6.670	0.0128	FDR<0.05
L1CAM	-6.667	0.0005	FDR<0.05
TLX2	-6.633	0.0044	FDR<0.05
ARSF	-6.626	0.0105	FDR<0.05
SEZ6L	-6.577	0.0007	FDR<0.05
CD24	-6.540	0.0002	FDR<0.05
CDK5R2	-6.527	0.0103	FDR<0.05
ACVR1C	-6.501	0.0000	FDR<0.05
AKAIN1	-6.491	0.0018	FDR<0.05
DRD2	-6.477	0.0009	FDR<0.05
PRLHR	-6.470	0.0053	FDR<0.05
RIMBP2	-6.469	0.0010	FDR<0.05
HAND2-AS1	-6.444	0.0004	FDR<0.05
ECE2	-6.432	0.0204	FDR<0.05
GPR158	-6.409	0.0056	FDR<0.05
MYH6	-6.402	0.0144	FDR<0.05
CACNA1E	-6.399	0.0066	FDR<0.05

BORCS7-ASMT	-6.385	0.0182	FDR<0.05
SLC8A3	-6.368	0.0094	FDR<0.05
AC063960.2	-6.364	0.0387	FDR<0.05
CA10	-6.347	0.0399	FDR<0.05
EPHA8	-6.324	0.0043	FDR<0.05
BMP7	-6.314	0.0004	FDR<0.05
RFX6	-6.314	0.0004	FDR<0.05
SYT13	-6.313	0.0161	FDR<0.05
KCNC1	-6.281	0.0139	FDR<0.05
GCGR	-6.265	0.0088	FDR<0.05
CFAP61	-6.253	0.0050	FDR<0.05
GABRQ	-6.230	0.0045	FDR<0.05
HEPACAM	-6.194	0.0011	FDR<0.05
CDH18	-6.188	0.0093	FDR<0.05
CADM2	-6.188	0.0144	FDR<0.05
PNMT	-6.106	0.0010	FDR<0.05
HCN1	-6.100	0.0245	FDR<0.05
BRINP3	-6.096	0.0039	FDR<0.05
ADCYAP1R1	-6.092	0.0018	FDR<0.05
LGI1	-6.090	0.0009	FDR<0.05
NWD2	-6.090	0.0412	FDR<0.05
CALY	-6.074	0.0017	FDR<0.05
CCDC85A	-6.068	0.0033	FDR<0.05
SEZ6	-6.050	0.0244	FDR<0.05
SYT5	-6.045	0.0008	FDR<0.05
CIDEA	-6.039	0.0016	FDR<0.05
ARFGEF3	-6.039	0.0001	FDR<0.05
SCG3	-6.025	0.0035	FDR<0.05
AP006333.2	-5.967	0.0254	FDR<0.05
TCEAL6	-5.916	0.0118	FDR<0.05
ELAVL4	-5.913	0.0001	FDR<0.05
ASIC4	-5.895	0.0077	FDR<0.05
TMEFF2	-5.887	0.0031	FDR<0.05
CARTPT	-5.887	0.0284	FDR<0.05
SAA2-SAA4	-5.878	0.0060	FDR<0.05
OPRM1	-5.849	0.0362	FDR<0.05
AC022893.2	-5.847	0.0137	FDR<0.05
PLIN1	-5.795	0.0000	FDR<0.05
EYA1	-5.776	0.0017	FDR<0.05
LRRC4B	-5.771	0.0016	FDR<0.05
UNC5A	-5.763	0.0017	FDR<0.05
AP004608.1	-5.760	0.0333	FDR<0.05
KLB	-5.758	0.0007	FDR<0.05
TAGLN3	-5.746	0.0070	FDR<0.05
COBL	-5.742	0.0000	FDR<0.05
MGAT4C	-5.728	0.0011	FDR<0.05

CAMK2B	-5.725	0.0002	FDR<0.05
CEND1	-5.717	0.0148	FDR<0.05
ABCC8	-5.682	0.0003	FDR<0.05
TMEM151B	-5.680	0.0042	FDR<0.05
GLRA3	-5.672	0.0338	FDR<0.05
FLRT1	-5.655	0.0050	FDR<0.05
DGKB	-5.614	0.0012	FDR<0.05
ZNF365	-5.605	0.0086	FDR<0.05
DCX	-5.601	0.0219	FDR<0.05
SYT1	-5.591	0.0003	FDR<0.05
DMRT2	-5.569	0.0026	FDR<0.05
PIANP	-5.564	0.0050	FDR<0.05
PTPRN	-5.559	0.0019	FDR<0.05
LHFPL4	-5.559	0.0161	FDR<0.05
IGFBPL1	-5.556	0.0030	FDR<0.05
GNG4	-5.523	0.0006	FDR<0.05
AL356218.2	-5.520	0.0018	FDR<0.05
LINC01595	-5.506	0.0001	FDR<0.05
AZGP1	-5.502	0.0001	FDR<0.05
CNTN3	-5.501	0.0009	FDR<0.05
NELL2	-5.495	0.0012	FDR<0.05
Mar-11	-5.469	0.0061	FDR<0.05
PPFIA2	-5.434	0.0008	FDR<0.05
SLC4A10	-5.414	0.0023	FDR<0.05
HRH3	-5.411	0.0010	FDR<0.05
COL20A1	-5.399	0.0010	FDR<0.05
APLP1	-5.369	0.0002	FDR<0.05
DDC	-5.358	0.0004	FDR<0.05
MAPT-IT1	-5.352	0.0418	FDR<0.05
OPRD1	-5.344	0.0244	FDR<0.05
SHISA9	-5.332	0.0451	FDR<0.05
PKHD1	-5.317	0.0216	FDR<0.05
KIF1A	-5.313	0.0050	FDR<0.05
NECAB1	-5.283	0.0458	FDR<0.05
SLC6A17	-5.279	0.0029	FDR<0.05
HAND2	-5.270	0.0111	FDR<0.05
KCNJ6	-5.231	0.0230	FDR<0.05
GRIK1	-5.231	0.0017	FDR<0.05
SNAP91	-5.212	0.0057	FDR<0.05
ZDHHC22	-5.212	0.0206	FDR<0.05
FAM163A	-5.188	0.0020	FDR<0.05
NTNG1	-5.185	0.0198	FDR<0.05
PPP1R17	-5.182	0.0351	FDR<0.05
DNAH17	-5.180	0.0002	FDR<0.05
EBF2	-5.177	0.0000	FDR<0.05
OLFM3	-5.170	0.0341	FDR<0.05

ITIH2	-5.167	0.0046	FDR<0.05
INA	-5.166	0.0023	FDR<0.05
SAA2	-5.148	0.0016	FDR<0.05
SLC8A2	-5.137	0.0137	FDR<0.05
HPCAL4	-5.123	0.0021	FDR<0.05
ATCAY	-5.116	0.0130	FDR<0.05
ACTL6B	-5.093	0.0267	FDR<0.05
EEF1AKMT4-ECE2	-5.088	0.0060	FDR<0.05
SRRM4	-5.068	0.0215	FDR<0.05
INSRR	-5.062	0.0010	FDR<0.05
MYOC	-5.061	0.0021	FDR<0.05
AC010478.1	-5.027	0.0464	FDR<0.05
NDST3	-5.021	0.0274	FDR<0.05
BMP8B	-5.019	0.0038	FDR<0.05
TMEM190	-5.005	0.0127	FDR<0.05
AP000781.2	-5.000	0.0173	FDR<0.05
XKR7	-4.994	0.0416	FDR<0.05
MYT1L	-4.991	0.0024	FDR<0.05
JPH3	-4.970	0.0023	FDR<0.05
PKIA	-4.965	0.0024	FDR<0.05
CHGB	-4.963	0.0005	FDR<0.05
SH3GL3	-4.959	0.0056	FDR<0.05
CRYBA2	-4.957	0.0105	FDR<0.05
NCAM1-AS1	-4.951	0.0408	FDR<0.05
POPDC3	-4.951	0.0046	FDR<0.05
PRPH	-4.943	0.0022	FDR<0.05
ATP1A3	-4.918	0.0007	FDR<0.05
CYP26C1	-4.906	0.0047	FDR<0.05
SPTB	-4.895	0.0001	FDR<0.05
LINC01230	-4.884	0.0338	FDR<0.05
LRRTM4	-4.882	0.0246	FDR<0.05
PROM1	-4.880	0.0053	FDR<0.05
MTMR7	-4.870	0.0050	FDR<0.05
CELF4	-4.866	0.0017	FDR<0.05
B3GAT1	-4.854	0.0013	FDR<0.05
LINC02607	-4.853	0.0239	FDR<0.05
KCNQ5	-4.835	0.0191	FDR<0.05
CHRNA3	-4.831	0.0185	FDR<0.05
KCNG1	-4.831	0.0155	FDR<0.05
GRIA2	-4.823	0.0289	FDR<0.05
DRC1	-4.805	0.0285	FDR<0.05
LINC02287	-4.805	0.0272	FDR<0.05
SRCIN1	-4.797	0.0024	FDR<0.05
SOX10	-4.789	0.0059	FDR<0.05
AC022239.1	-4.786	0.0230	FDR<0.05
DLX1	-4.775	0.0038	FDR<0.05

GATA3-AS1	-4.731	0.0387	FDR<0.05
APC2	-4.682	0.0026	FDR<0.05
PLPPR4	-4.678	0.0357	FDR<0.05
AC126755.6	-4.674	0.0000	FDR<0.05
CAMKV	-4.666	0.0427	FDR<0.05
INSM2	-4.661	0.0158	FDR<0.05
KIAA0319	-4.650	0.0098	FDR<0.05
KCNH1	-4.642	0.0262	FDR<0.05
PMP2	-4.636	0.0123	FDR<0.05
AL450267.2	-4.632	0.0317	FDR<0.05
NSG2	-4.610	0.0012	FDR<0.05
NTRK1	-4.566	0.0016	FDR<0.05
MYCN	-4.544	0.0001	FDR<0.05
TNFSF18	-4.540	0.0032	FDR<0.05
AGMO	-4.534	0.0267	FDR<0.05
AC134312.1	-4.528	0.0276	FDR<0.05
VWDE	-4.526	0.0444	FDR<0.05
SVOP	-4.520	0.0097	FDR<0.05
FFAR3	-4.514	0.0091	FDR<0.05
TCEAL5	-4.508	0.0139	FDR<0.05
RASEF	-4.502	0.0041	FDR<0.05
AC000093.1	-4.497	0.0076	FDR<0.05
AC015961.1	-4.458	0.0310	FDR<0.05
PCDH11X	-4.455	0.0174	FDR<0.05
SPX	-4.454	0.0002	FDR<0.05
VIT	-4.454	0.0146	FDR<0.05
DPP6	-4.447	0.0029	FDR<0.05
ATP1A2	-4.432	0.0006	FDR<0.05
Mar-04	-4.429	0.0150	FDR<0.05
C2orf72	-4.428	0.0009	FDR<0.05
AC107373.2	-4.426	0.0250	FDR<0.05
NRXN3	-4.419	0.0029	FDR<0.05
TNMD	-4.419	0.0104	FDR<0.05
LINGO4	-4.413	0.0149	FDR<0.05
CNGB1	-4.386	0.0467	FDR<0.05
NMNAT2	-4.355	0.0002	FDR<0.05
DDX25	-4.317	0.0005	FDR<0.05
SLC38A11	-4.314	0.0038	FDR<0.05
C4orf50	-4.295	0.0165	FDR<0.05
AMPH	-4.287	0.0014	FDR<0.05
KRT73	-4.284	0.0047	FDR<0.05
HCRTR1	-4.283	0.0174	FDR<0.05
TAFA3	-4.275	0.0407	FDR<0.05
AL161630.1	-4.274	0.0462	FDR<0.05
NLGN4X	-4.256	0.0000	FDR<0.05
SCN3B	-4.256	0.0014	FDR<0.05

MIAT	-4.253	0.0026	FDR<0.05
LRRTM1	-4.230	0.0500	FDR<0.05
PTPRR	-4.226	0.0190	FDR<0.05
CYP26A1	-4.210	0.0063	FDR<0.05
CACNG4	-4.202	0.0094	FDR<0.05
RET	-4.167	0.0001	FDR<0.05
CCSER1	-4.164	0.0026	FDR<0.05
BMP5	-4.164	0.0436	FDR<0.05
GRIK2	-4.157	0.0063	FDR<0.05
MAGEE2	-4.144	0.0041	FDR<0.05
RXRG	-4.129	0.0006	FDR<0.05
TCEAL2	-4.100	0.0001	FDR<0.05
CHST8	-4.098	0.0238	FDR<0.05
ADD2	-4.098	0.0016	FDR<0.05
SFRP5	-4.096	0.0219	FDR<0.05
PCSK1N	-4.095	0.0244	FDR<0.05
AL049749.1	-4.089	0.0492	FDR<0.05
S100B	-4.080	0.0000	FDR<0.05
MAB21L2	-4.077	0.0311	FDR<0.05
GPC5	-4.056	0.0346	FDR<0.05
PROKR1	-4.041	0.0225	FDR<0.05
KIR2DL4	-4.019	0.0006	FDR<0.05
USH2A	-4.019	0.0198	FDR<0.05
CFAP65	-3.998	0.0129	FDR<0.05
GRIK3	-3.993	0.0032	FDR<0.05
LRRTM2	-3.991	0.0085	FDR<0.05
EFHC2	-3.958	0.0060	FDR<0.05
PCSK1	-3.954	0.0073	FDR<0.05
AC061975.7	-3.951	0.0214	FDR<0.05
GNG3	-3.942	0.0013	FDR<0.05
SLC4A4	-3.941	0.0001	FDR<0.05
MTCL1	-3.938	0.0006	FDR<0.05
KCNK12	-3.921	0.0023	FDR<0.05
NRXN1	-3.902	0.0423	FDR<0.05
JPH4	-3.886	0.0006	FDR<0.05
SGCG	-3.884	0.0249	FDR<0.05
FRMD1	-3.883	0.0122	FDR<0.05
SLC16A12	-3.879	0.0003	FDR<0.05
KCNK9	-3.875	0.0159	FDR<0.05
CHRNB2	-3.868	0.0012	FDR<0.05
FLRT3	-3.866	0.0013	FDR<0.05
BTBD16	-3.853	0.0225	FDR<0.05
AC022424.1	-3.848	0.0251	FDR<0.05
STRA6	-3.847	0.0048	FDR<0.05
EYA4	-3.846	0.0336	FDR<0.05
CAMK2A	-3.842	0.0072	FDR<0.05

LINC02458	-3.841	0.0029	FDR<0.05
TNN	-3.833	0.0033	FDR<0.05
AC006511.5	-3.828	0.0253	FDR<0.05
NEFL	-3.819	0.0075	FDR<0.05
IL13RA2	-3.819	0.0093	FDR<0.05
SCN3A	-3.816	0.0051	FDR<0.05
TMEM45B	-3.814	0.0043	FDR<0.05
CAV1	-3.795	0.0000	FDR<0.05
RNF175	-3.793	0.0054	FDR<0.05
NXF2B	-3.785	0.0201	FDR<0.05
TOX3	-3.783	0.0199	FDR<0.05
AL845331.2	-3.776	0.0240	FDR<0.05
INSC	-3.763	0.0240	FDR<0.05
ITIH1	-3.758	0.0104	FDR<0.05
AL136964.1	-3.752	0.0372	FDR<0.05
SLCO1C1	-3.747	0.0240	FDR<0.05
LPL	-3.743	0.0000	FDR<0.05
CACNA1B	-3.739	0.0382	FDR<0.05
ADGRB3	-3.732	0.0015	FDR<0.05
PRIMA1	-3.705	0.0115	FDR<0.05
SCN5A	-3.705	0.0028	FDR<0.05
B3GALT1	-3.702	0.0258	FDR<0.05
B3GALT5-AS1	-3.702	0.0154	FDR<0.05
CDH22	-3.694	0.0339	FDR<0.05
AC093899.2	-3.685	0.0194	FDR<0.05
PRG4	-3.680	0.0030	FDR<0.05
APCDD1L	-3.677	0.0104	FDR<0.05
NEURL1	-3.670	0.0152	FDR<0.05
KCTD16	-3.666	0.0149	FDR<0.05
CYP4A11	-3.659	0.0225	FDR<0.05
GALNT8	-3.653	0.0039	FDR<0.05
AC007009.1	-3.649	0.0068	FDR<0.05
RNASEK-C17orf49	-3.634	0.0005	FDR<0.05
CHST9	-3.633	0.0306	FDR<0.05
PCOLCE2	-3.626	0.0012	FDR<0.05
KCNB1	-3.626	0.0019	FDR<0.05
AL591806.4	-3.589	0.0184	FDR<0.05
PI16	-3.587	0.0005	FDR<0.05
SELE	-3.585	0.0001	FDR<0.05
CLUL1	-3.585	0.0176	FDR<0.05
STMN4	-3.582	0.0097	FDR<0.05
KLHL32	-3.581	0.0030	FDR<0.05
LRRC10B	-3.561	0.0023	FDR<0.05
FREM2	-3.557	0.0252	FDR<0.05
SLC10A6	-3.557	0.0083	FDR<0.05
PCDHGB6	-3.548	0.0320	FDR<0.05

NXPH4	-3.537	0.0069	FDR<0.05
SYCE1	-3.536	0.0013	FDR<0.05
SPSB4	-3.533	0.0076	FDR<0.05
PRSS50_1	-3.532	0.0035	FDR<0.05
CAMK4	-3.531	0.0017	FDR<0.05
S100A1	-3.516	0.0011	FDR<0.05
AC005740.5	-3.504	0.0467	FDR<0.05
TTLL10-AS1	-3.502	0.0324	FDR<0.05
ZMAT4	-3.498	0.0497	FDR<0.05
TLCD3B	-3.496	0.0039	FDR<0.05
RIMS4	-3.495	0.0062	FDR<0.05
ITGB3	-3.489	0.0031	FDR<0.05
ACTC1	-3.482	0.0420	FDR<0.05
LINC00632	-3.460	0.0008	FDR<0.05
THSD7B	-3.459	0.0106	FDR<0.05
GRIN2A	-3.454	0.0050	FDR<0.05
LINC02182	-3.431	0.0290	FDR<0.05
MME	-3.414	0.0016	FDR<0.05
CHRM4	-3.414	0.0239	FDR<0.05
AC110619.1	-3.400	0.0280	FDR<0.05
PDE10A	-3.396	0.0005	FDR<0.05
TCEAL7	-3.392	0.0005	FDR<0.05
AL022337.1	-3.386	0.0172	FDR<0.05
TRHDE-AS1	-3.382	0.0175	FDR<0.05
AQP7	-3.382	0.0003	FDR<0.05
SUSD4	-3.373	0.0010	FDR<0.05
HCRTR2	-3.371	0.0422	FDR<0.05
PLCH2	-3.367	0.0006	FDR<0.05
B3GALT5	-3.364	0.0401	FDR<0.05
PRDM8	-3.347	0.0007	FDR<0.05
RGS17	-3.333	0.0325	FDR<0.05
AL162574.1	-3.324	0.0315	FDR<0.05
LINC02714	-3.319	0.0128	FDR<0.05
HCAR2	-3.317	0.0104	FDR<0.05
AC107464.1	-3.307	0.0107	FDR<0.05
TDRD12	-3.307	0.0031	FDR<0.05
HSPE1-MOB4	-3.300	0.0008	FDR<0.05
AC005064.1	-3.299	0.0046	FDR<0.05
NDNF	-3.298	0.0153	FDR<0.05
HPCA	-3.294	0.0205	FDR<0.05
SFRP2	-3.292	0.0114	FDR<0.05
AKR1C2	-3.284	0.0003	FDR<0.05
SOSTDC1	-3.284	0.0060	FDR<0.05
KANK4	-3.283	0.0042	FDR<0.05
RIPPLY2	-3.283	0.0318	FDR<0.05
PDIA2	-3.279	0.0040	FDR<0.05

RETREG1	-3.276	0.0017	FDR<0.05
FAM167A	-3.276	0.0186	FDR<0.05
SLC19A3	-3.263	0.0038	FDR<0.05
CCDC181	-3.260	0.0101	FDR<0.05
MAPT	-3.257	0.0003	FDR<0.05
GPM6B	-3.250	0.0013	FDR<0.05
SEMA3E	-3.247	0.0464	FDR<0.05
HMGCLL1	-3.241	0.0043	FDR<0.05
SOX2	-3.234	0.0423	FDR<0.05
SLC35F3	-3.234	0.0107	FDR<0.05
SERTAD4	-3.231	0.0029	FDR<0.05
BCAN	-3.229	0.0200	FDR<0.05
ENTPD3	-3.225	0.0059	FDR<0.05
SERTM1	-3.224	0.0165	FDR<0.05
AL356740.3	-3.218	0.0209	FDR<0.05
FABP5	-3.214	0.0002	FDR<0.05
AC011008.1	-3.208	0.0340	FDR<0.05
FAM162B	-3.199	0.0011	FDR<0.05
KRT19	-3.188	0.0197	FDR<0.05
TUBB2B	-3.185	0.0011	FDR<0.05
PRRT4	-3.179	0.0071	FDR<0.05
AC018865.3	-3.161	0.0055	FDR<0.05
TGFBR3L	-3.160	0.0302	FDR<0.05
SGIP1	-3.157	0.0026	FDR<0.05
FRMPD3	-3.155	0.0325	FDR<0.05
AC006058.1	-3.139	0.0357	FDR<0.05
HUNK	-3.139	0.0005	FDR<0.05
GABRB3	-3.137	0.0005	FDR<0.05
PCDH10	-3.132	0.0492	FDR<0.05
GAL	-3.130	0.0486	FDR<0.05
SPEG	-3.123	0.0033	FDR<0.05
AC055839.2	-3.118	0.0022	FDR<0.05
SCN1A-AS1	-3.115	0.0334	FDR<0.05
PPP1R1C	-3.111	0.0039	FDR<0.05
SLC26A4-AS1	-3.099	0.0247	FDR<0.05
AC011246.1	-3.088	0.0145	FDR<0.05
NOX4	-3.066	0.0009	FDR<0.05
SNAP25	-3.064	0.0218	FDR<0.05
HS6ST2	-3.054	0.0040	FDR<0.05
KRT72	-3.049	0.0304	FDR<0.05
PPFIA4	-3.045	0.0017	FDR<0.05
SCN9A	-3.041	0.0057	FDR<0.05
SV2C	-3.035	0.0449	FDR<0.05
CACNA2D3	-3.032	0.0073	FDR<0.05
AL021396.1	-3.030	0.0147	FDR<0.05
ADAM12	-3.027	0.0053	FDR<0.05

CDH19	-3.025	0.0276	FDR<0.05
AC093523.1	-3.015	0.0310	FDR<0.05
GABRP	-3.013	0.0325	FDR<0.05
AL160286.2	-3.005	0.0099	FDR<0.05
AC005696.4	-2.998	0.0248	FDR<0.05
AL136090.2	-2.995	0.0235	FDR<0.05
FAIM2	-2.977	0.0354	FDR<0.05
FAXC	-2.975	0.0071	FDR<0.05
AL391807.1	-2.954	0.0063	FDR<0.05
AJ239328.1	-2.950	0.0208	FDR<0.05
CNIH2	-2.948	0.0083	FDR<0.05
KIF5A	-2.944	0.0428	FDR<0.05
LINC02268	-2.942	0.0368	FDR<0.05
POSTN	-2.937	0.0082	FDR<0.05
SPATA22	-2.933	0.0156	FDR<0.05
DCLK3	-2.932	0.0253	FDR<0.05
CDH8	-2.925	0.0390	FDR<0.05
ARHGDIG	-2.924	0.0208	FDR<0.05
SDK2	-2.923	0.0024	FDR<0.05
LINC01018	-2.910	0.0071	FDR<0.05
RADIL	-2.903	0.0030	FDR<0.05
B4GALNT4	-2.902	0.0093	FDR<0.05
AC022784.1	-2.898	0.0148	FDR<0.05
EPHX4	-2.893	0.0038	FDR<0.05
BMPER	-2.891	0.0009	FDR<0.05
PRUNE2	-2.885	0.0046	FDR<0.05
ENPEP	-2.882	0.0000	FDR<0.05
RBM24	-2.876	0.0231	FDR<0.05
MEOX1	-2.874	0.0386	FDR<0.05
TAS2R20	-2.873	0.0017	FDR<0.05
TRPC6	-2.870	0.0000	FDR<0.05
AC114284.1	-2.869	0.0494	FDR<0.05
PTPRZ1	-2.869	0.0141	FDR<0.05
AC073957.3	-2.868	0.0006	FDR<0.05
IL6	-2.861	0.0232	FDR<0.05
GREM1	-2.858	0.0090	FDR<0.05
TNFAIP6	-2.854	0.0402	FDR<0.05
GATA2	-2.852	0.0016	FDR<0.05
TTLL7	-2.851	0.0005	FDR<0.05
AC009549.1	-2.850	0.0076	FDR<0.05
ZCCHC12	-2.826	0.0025	FDR<0.05
C14orf39	-2.822	0.0370	FDR<0.05
PPM1J	-2.821	0.0051	FDR<0.05
GPR27	-2.811	0.0217	FDR<0.05
CELA2A	-2.805	0.0310	FDR<0.05
CLIC6	-2.804	0.0244	FDR<0.05

CNTNAP3	-2.802	0.0078	FDR<0.05
GJC3	-2.790	0.0207	FDR<0.05
RGS4	-2.784	0.0098	FDR<0.05
GDF10	-2.781	0.0244	FDR<0.05
C1QL1	-2.775	0.0437	FDR<0.05
BRINP1	-2.773	0.0228	FDR<0.05
ADH1C	-2.773	0.0026	FDR<0.05
TMEM132C	-2.767	0.0009	FDR<0.05
GUCY1B1	-2.766	0.0000	FDR<0.05
AC011498.4	-2.763	0.0077	FDR<0.05
SMAD9	-2.763	0.0005	FDR<0.05
MAP2	-2.756	0.0007	FDR<0.05
GALNT14	-2.752	0.0022	FDR<0.05
MAGED4B	-2.748	0.0157	FDR<0.05
AK5	-2.739	0.0454	FDR<0.05
ZNF534	-2.738	0.0455	FDR<0.05
IGSF9B	-2.737	0.0054	FDR<0.05
RIMS3	-2.731	0.0022	FDR<0.05
CADM3	-2.730	0.0140	FDR<0.05
ADH4	-2.727	0.0076	FDR<0.05
SCGB1D2	-2.727	0.0133	FDR<0.05
ABCF2_1	-2.724	0.0399	FDR<0.05
PCLO	-2.722	0.0002	FDR<0.05
PNMA2	-2.718	0.0019	FDR<0.05
EFS	-2.713	0.0012	FDR<0.05
TPPP3	-2.710	0.0033	FDR<0.05
LGI4	-2.702	0.0027	FDR<0.05
JAKMIP1	-2.700	0.0317	FDR<0.05
HP	-2.692	0.0086	FDR<0.05
AC093326.1	-2.692	0.0488	FDR<0.05
AC092490.1	-2.690	0.0110	FDR<0.05
GRIK5	-2.687	0.0438	FDR<0.05
PCDH9	-2.685	0.0174	FDR<0.05
SORCS1	-2.684	0.0145	FDR<0.05
RNF165	-2.681	0.0030	FDR<0.05
FOSB	-2.673	0.0067	FDR<0.05
KIF26A	-2.668	0.0014	FDR<0.05
LYPD6	-2.667	0.0484	FDR<0.05
EPHA3	-2.667	0.0319	FDR<0.05
SLC1A1	-2.664	0.0003	FDR<0.05
PKP1	-2.662	0.0141	FDR<0.05
AKR1C1	-2.660	0.0015	FDR<0.05
AP005136.3	-2.656	0.0041	FDR<0.05
CDHR1	-2.654	0.0044	FDR<0.05
STAC	-2.653	0.0067	FDR<0.05
CDH6	-2.650	0.0099	FDR<0.05

GYG2	-2.649	0.0063	FDR<0.05
CXCL13	-2.648	0.0498	FDR<0.05
MAP1LC3C	-2.644	0.0231	FDR<0.05
SAMD5	-2.643	0.0065	FDR<0.05
ADGRF4	-2.636	0.0355	FDR<0.05
SLC6A4	-2.635	0.0338	FDR<0.05
ITIH5	-2.632	0.0125	FDR<0.05
NPIPB8	-2.631	0.0068	FDR<0.05
TMEM256-PLSCR3	-2.624	0.0367	FDR<0.05
SORBS1	-2.624	0.0009	FDR<0.05
AL133482.1	-2.622	0.0472	FDR<0.05
AL109741.1	-2.620	0.0451	FDR<0.05
MEOX2	-2.610	0.0138	FDR<0.05
RP1L1	-2.608	0.0379	FDR<0.05
PCDHB6	-2.607	0.0096	FDR<0.05
CD36	-2.605	0.0052	FDR<0.05
TMSB15A	-2.602	0.0005	FDR<0.05
MLPH	-2.601	0.0035	FDR<0.05
GNAO1	-2.599	0.0035	FDR<0.05
RAMP2-AS1	-2.598	0.0006	FDR<0.05
AK4	-2.594	0.0023	FDR<0.05
PAK3	-2.594	0.0089	FDR<0.05
WIF1	-2.593	0.0286	FDR<0.05
AC105383.1	-2.592	0.0494	FDR<0.05
IGF2-AS	-2.586	0.0351	FDR<0.05
PDZD2	-2.579	0.0003	FDR<0.05
TDRD9	-2.578	0.0019	FDR<0.05
CYP27C1	-2.577	0.0304	FDR<0.05
MAGED4	-2.576	0.0247	FDR<0.05
TTK	-2.573	0.0024	FDR<0.05
DAB1	-2.572	0.0156	FDR<0.05
AC012065.1	-2.562	0.0048	FDR<0.05
KCNH8	-2.560	0.0212	FDR<0.05
ISLR2	-2.560	0.0157	FDR<0.05
RASGRF2-AS1	-2.543	0.0164	FDR<0.05
SCHIP1	-2.537	0.0016	FDR<0.05
BEX1	-2.536	0.0145	FDR<0.05
LGALS12	-2.530	0.0061	FDR<0.05
FGF14	-2.526	0.0294	FDR<0.05
RAMP1	-2.520	0.0174	FDR<0.05
ALK	-2.520	0.0093	FDR<0.05
DYNC1I1	-2.516	0.0046	FDR<0.05
AC007744.1	-2.507	0.0034	FDR<0.05
FABP4	-2.500	0.0026	FDR<0.05
GPR137C	-2.499	0.0014	FDR<0.05
KLK10	-2.488	0.0474	FDR<0.05

PON3	-2.488	0.0029	FDR<0.05
AGBL4	-2.487	0.0041	FDR<0.05
CXXC4	-2.487	0.0044	FDR<0.05
EGR2	-2.482	0.0053	FDR<0.05
PDE3B	-2.480	0.0088	FDR<0.05
SORCS3	-2.471	0.0343	FDR<0.05
AC104088.3	-2.461	0.0430	FDR<0.05
SPAG16-DT	-2.455	0.0274	FDR<0.05
TTBK1	-2.449	0.0145	FDR<0.05
KLK3	-2.444	0.0190	FDR<0.05
ADRB1	-2.443	0.0110	FDR<0.05
PWWP3B	-2.437	0.0249	FDR<0.05
PCDHB4	-2.421	0.0006	FDR<0.05
RBP4	-2.419	0.0498	FDR<0.05
PLCB4	-2.416	0.0149	FDR<0.05
GRM2	-2.414	0.0143	FDR<0.05
KDR	-2.412	0.0000	FDR<0.05
PCDHB3	-2.405	0.0236	FDR<0.05
MECOM	-2.401	0.0040	FDR<0.05
TRIM9	-2.400	0.0100	FDR<0.05
SLC38A3	-2.395	0.0242	FDR<0.05
KCNIP2	-2.391	0.0018	FDR<0.05
FGF1	-2.391	0.0014	FDR<0.05
NAT8L	-2.385	0.0179	FDR<0.05
AC079949.2	-2.385	0.0274	FDR<0.05
PLCD4	-2.384	0.0084	FDR<0.05
AC090337.1	-2.376	0.0374	FDR<0.05
BMP3	-2.373	0.0429	FDR<0.05
ANGPT1	-2.369	0.0017	FDR<0.05
AC092329.1	-2.361	0.0310	FDR<0.05
NUDT10	-2.357	0.0009	FDR<0.05
CELSR3	-2.354	0.0038	FDR<0.05
AC004877.1	-2.352	0.0100	FDR<0.05
SGCA	-2.351	0.0274	FDR<0.05
PAK5	-2.348	0.0000	FDR<0.05
CALCRL	-2.347	0.0000	FDR<0.05
NAP1L3	-2.345	0.0004	FDR<0.05
OGDHL	-2.343	0.0234	FDR<0.05
ARHGEF4	-2.342	0.0089	FDR<0.05
CD300LG	-2.342	0.0023	FDR<0.05
CXCL8	-2.342	0.0130	FDR<0.05
MAP7D2	-2.339	0.0099	FDR<0.05
LINC00484	-2.332	0.0279	FDR<0.05
DNAJC6	-2.329	0.0000	FDR<0.05
DCLK1	-2.328	0.0297	FDR<0.05
PLIN4	-2.312	0.0027	FDR<0.05

LINC00598	-2.306	0.0033	FDR<0.05
CDH4	-2.304	0.0389	FDR<0.05
UNC79	-2.303	0.0410	FDR<0.05
TIMP4	-2.302	0.0025	FDR<0.05
SQLE	-2.302	0.0110	FDR<0.05
AC104083.1	-2.301	0.0033	FDR<0.05
MYRIP	-2.301	0.0051	FDR<0.05
BTNL9	-2.300	0.0026	FDR<0.05
ITM2A	-2.299	0.0011	FDR<0.05
MYO1B	-2.296	0.0000	FDR<0.05
LVRN	-2.294	0.0264	FDR<0.05
AP000873.3	-2.294	0.0087	FDR<0.05
KL	-2.291	0.0443	FDR<0.05
GPLD1	-2.291	0.0017	FDR<0.05
SSTR2	-2.291	0.0034	FDR<0.05
IQCA1	-2.289	0.0268	FDR<0.05
GPR83	-2.288	0.0148	FDR<0.05
MELTF	-2.287	0.0187	FDR<0.05
TSPAN2	-2.287	0.0056	FDR<0.05
DGAT2	-2.275	0.0093	FDR<0.05
PLAG1	-2.273	0.0046	FDR<0.05
MLLT11	-2.267	0.0028	FDR<0.05
PKP2	-2.265	0.0084	FDR<0.05
SMOC1	-2.264	0.0156	FDR<0.05
STBD1	-2.261	0.0092	FDR<0.05
TAFA1	-2.257	0.0408	FDR<0.05
AC130466.1	-2.251	0.0294	FDR<0.05
LINC02202	-2.251	0.0093	FDR<0.05
NAP1L2	-2.247	0.0002	FDR<0.05
USH1C	-2.247	0.0426	FDR<0.05
AGAP11	-2.244	0.0067	FDR<0.05
SEMA5A	-2.236	0.0006	FDR<0.05
C9orf153	-2.232	0.0232	FDR<0.05
EGR3	-2.232	0.0141	FDR<0.05
KCNJ12	-2.230	0.0047	FDR<0.05
MAL2	-2.229	0.0194	FDR<0.05
AC010655.2	-2.225	0.0087	FDR<0.05
AC004540.2	-2.223	0.0044	FDR<0.05
AC090164.5	-2.221	0.0451	FDR<0.05
LIMS4	-2.219	0.0143	FDR<0.05
RNF112	-2.216	0.0222	FDR<0.05
ABCG4	-2.210	0.0037	FDR<0.05
RAP1GAP2	-2.208	0.0038	FDR<0.05
ECT2	-2.208	0.0021	FDR<0.05
AC006249.1	-2.205	0.0416	FDR<0.05
SLC1A2	-2.198	0.0012	FDR<0.05

ADAMTS5	-2.191	0.0058	FDR<0.05
ASPG	-2.186	0.0045	FDR<0.05
IGF2	-2.185	0.0034	FDR<0.05
IQSEC3	-2.184	0.0272	FDR<0.05
AC115618.1	-2.184	0.0204	FDR<0.05
DNAH10	-2.183	0.0310	FDR<0.05
IQCJ-SCHIP1	-2.181	0.0024	FDR<0.05
RHOXF1-AS1	-2.180	0.0266	FDR<0.05
IP6K3	-2.179	0.0360	FDR<0.05
PCDHGC3	-2.179	0.0413	FDR<0.05
FOXF2	-2.165	0.0271	FDR<0.05
IGSF3	-2.164	0.0003	FDR<0.05
PABPN1L	-2.163	0.0225	FDR<0.05
TEK	-2.163	0.0000	FDR<0.05
PHGDH	-2.159	0.0195	FDR<0.05
GREM2	-2.155	0.0098	FDR<0.05
TUBA3FP	-2.152	0.0297	FDR<0.05
ART3	-2.149	0.0479	FDR<0.05
PCDH18	-2.148	0.0040	FDR<0.05
FHL1	-2.144	0.0012	FDR<0.05
GOLGA8S	-2.141	0.0143	FDR<0.05
PTGS2	-2.139	0.0035	FDR<0.05
KIRREL3	-2.139	0.0348	FDR<0.05
RHPN2	-2.138	0.0148	FDR<0.05
BEGAIN	-2.136	0.0096	FDR<0.05
THRSP	-2.135	0.0293	FDR<0.05
PLA2G4A	-2.135	0.0030	FDR<0.05
MAPK15	-2.127	0.0389	FDR<0.05
CXCL9	-2.125	0.0137	FDR<0.05
SUSD5	-2.121	0.0308	FDR<0.05
AC103702.1	-2.121	0.0247	FDR<0.05
SMTNL2	-2.114	0.0497	FDR<0.05
ACKR3	-2.113	0.0118	FDR<0.05
CREB5	-2.108	0.0007	FDR<0.05
KCNAB1	-2.108	0.0490	FDR<0.05
RAB6B	-2.108	0.0032	FDR<0.05
ADARB2	-2.099	0.0170	FDR<0.05
RGS5	-2.097	0.0031	FDR<0.05
LINC01933	-2.094	0.0299	FDR<0.05
TMEM179	-2.093	0.0319	FDR<0.05
WDR17	-2.091	0.0060	FDR<0.05
AC061975.8	-2.090	0.0091	FDR<0.05
KLHL13	-2.089	0.0004	FDR<0.05
MSANTD3-TMEFF1	-2.087	0.0119	FDR<0.05
SERPINI1	-2.087	0.0044	FDR<0.05

KLHL23	-2.084	0.0009	FDR<0.05
ARHGEF16	-2.083	0.0033	FDR<0.05
AC078795.1	-2.083	0.0114	FDR<0.05
EML5	-2.081	0.0029	FDR<0.05
TMEM145	-2.081	0.0113	FDR<0.05
AP006621.2	-2.079	0.0193	FDR<0.05
EFNB2	-2.075	0.0000	FDR<0.05
ENPP6	-2.073	0.0380	FDR<0.05
CORO2A	-2.073	0.0150	FDR<0.05
IL33	-2.070	0.0011	FDR<0.05
FOXC2	-2.069	0.0157	FDR<0.05
AF131215.5	-2.068	0.0036	FDR<0.05
SPARCL1	-2.068	0.0002	FDR<0.05
SCN4A	-2.065	0.0027	FDR<0.05
CNTFR	-2.055	0.0410	FDR<0.05
ANOS1	-2.053	0.0047	FDR<0.05
AC007285.1	-2.051	0.0340	FDR<0.05
ANO1	-2.050	0.0070	FDR<0.05
PDE4D	-2.050	0.0029	FDR<0.05
SDK1	-2.049	0.0035	FDR<0.05
PPARG	-2.042	0.0023	FDR<0.05
GUCY1A1	-2.039	0.0033	FDR<0.05
PRDM16	-2.038	0.0088	FDR<0.05
HDAC9	-2.037	0.0106	FDR<0.05
AL513165.1	-2.034	0.0073	FDR<0.05
SERTAD4-AS1	-2.025	0.0422	FDR<0.05
AC007743.1	-2.025	0.0204	FDR<0.05
AC124312.1	-2.024	0.0131	FDR<0.05
AL121820.3	-2.024	0.0194	FDR<0.05
FBXL16	-2.024	0.0370	FDR<0.05
TCIM	-2.020	0.0022	FDR<0.05
AP3B2	-2.019	0.0079	FDR<0.05
AL583785.1	-2.018	0.0417	FDR<0.05
KLHL33	-2.018	0.0123	FDR<0.05
GPRIN1	-2.014	0.0350	FDR<0.05
UNC5B	-2.008	0.0008	FDR<0.05
SEMA6D	-2.007	0.0019	FDR<0.05
IL12A	-2.006	0.0465	FDR<0.05
PARM1	-2.002	0.0024	FDR<0.05
AC068547.1	-2.001	0.0218	FDR<0.05
SMIM11A	2.000	0.0040	FDR<0.05
RNU4-62P	2.001	0.0031	FDR<0.05
LTB	2.001	0.0020	FDR<0.05
AC067930.4	2.002	0.0171	FDR<0.05
PCOLCE	2.003	0.0044	FDR<0.05
APOBEC2	2.004	0.0149	FDR<0.05

P2RX5	2.004	0.0128	FDR<0.05
TSGA13	2.006	0.0296	FDR<0.05
AC005081.1	2.008	0.0063	FDR<0.05
TAS1R1	2.009	0.0243	FDR<0.05
CATSPERZ	2.010	0.0185	FDR<0.05
CRB3	2.015	0.0101	FDR<0.05
LINC01730	2.019	0.0168	FDR<0.05
CD19	2.026	0.0090	FDR<0.05
AC009630.3	2.029	0.0237	FDR<0.05
AC245041.2	2.031	0.0114	FDR<0.05
AC009690.2	2.035	0.0222	FDR<0.05
FOLR3	2.037	0.0493	FDR<0.05
BX546450.2	2.043	0.0420	FDR<0.05
MCEMP1	2.049	0.0370	FDR<0.05
CDH15	2.049	0.0382	FDR<0.05
ZNF114	2.050	0.0489	FDR<0.05
AC020912.1	2.054	0.0158	FDR<0.05
AP001160.1	2.054	0.0029	FDR<0.05
AP002812.5	2.055	0.0055	FDR<0.05
SLC30A3	2.063	0.0158	FDR<0.05
THRB-IT1	2.065	0.0257	FDR<0.05
AC010300.1	2.066	0.0010	FDR<0.05
AC073263.1	2.075	0.0093	FDR<0.05
MADCAM1	2.077	0.0004	FDR<0.05
TLE6	2.079	0.0059	FDR<0.05
AC010997.5	2.082	0.0006	FDR<0.05
P2RX5-TAX1BP3	2.087	0.0118	FDR<0.05
AC105460.1	2.090	0.0344	FDR<0.05
ZACN	2.091	0.0031	FDR<0.05
AC090617.3	2.094	0.0017	FDR<0.05
APOA1	2.095	0.0256	FDR<0.05
PCP2	2.095	0.0041	FDR<0.05
GADD45G	2.099	0.0000	FDR<0.05
MSC	2.107	0.0002	FDR<0.05
AC012186.2	2.108	0.0274	FDR<0.05
AC064836.4	2.117	0.0073	FDR<0.05
H1FNT	2.124	0.0240	FDR<0.05
SH2D3A	2.125	0.0002	FDR<0.05
CXorf65	2.131	0.0071	FDR<0.05
AP002807.1	2.133	0.0013	FDR<0.05
C9orf16	2.138	0.0000	FDR<0.05
AC011447.3	2.141	0.0000	FDR<0.05
AC004528.2	2.142	0.0104	FDR<0.05
KRT18	2.143	0.0199	FDR<0.05
CDHR5	2.144	0.0434	FDR<0.05
AC006059.5	2.147	0.0332	FDR<0.05

ACSM3	2.152	0.0031	FDR<0.05
COL9A2	2.154	0.0029	FDR<0.05
AC034102.4	2.156	0.0347	FDR<0.05
HPX	2.166	0.0319	FDR<0.05
AC002401.4	2.167	0.0286	FDR<0.05
TRIM31	2.167	0.0460	FDR<0.05
PTPRCAP	2.169	0.0003	FDR<0.05
AC093503.2	2.170	0.0056	FDR<0.05
KCNIP3	2.175	0.0008	FDR<0.05
PRMT5-AS1	2.184	0.0368	FDR<0.05
PYCARD-AS1	2.186	0.0019	FDR<0.05
LY6D	2.187	0.0023	FDR<0.05
IL17B	2.190	0.0011	FDR<0.05
AC004585.1	2.190	0.0188	FDR<0.05
LNCOG	2.191	0.0287	FDR<0.05
SULT2B1	2.196	0.0013	FDR<0.05
AC016949.1	2.205	0.0068	FDR<0.05
MT-TA	2.208	0.0101	FDR<0.05
DUOXA1	2.209	0.0384	FDR<0.05
LINC00311	2.213	0.0034	FDR<0.05
RN7SL233P	2.219	0.0189	FDR<0.05
LINC01497	2.220	0.0023	FDR<0.05
LCN12	2.235	0.0004	FDR<0.05
LINC00685	2.236	0.0005	FDR<0.05
HTRA4	2.237	0.0106	FDR<0.05
AC004882.3	2.239	0.0110	FDR<0.05
TNNI2	2.241	0.0000	FDR<0.05
SCT	2.244	0.0122	FDR<0.05
ENO3	2.244	0.0002	FDR<0.05
AC007448.4	2.246	0.0002	FDR<0.05
CEMP1	2.247	0.0054	FDR<0.05
MIF-AS1	2.252	0.0020	FDR<0.05
AC011511.3	2.255	0.0181	FDR<0.05
RNU1-106P	2.256	0.0368	FDR<0.05
S100A14	2.260	0.0485	FDR<0.05
ZBP1	2.262	0.0057	FDR<0.05
SSTR5-AS1	2.262	0.0313	FDR<0.05
PNMA5	2.263	0.0225	FDR<0.05
AC005785.1	2.263	0.0008	FDR<0.05
NUP210L	2.264	0.0360	FDR<0.05
TFR2	2.266	0.0071	FDR<0.05
DLX4	2.271	0.0230	FDR<0.05
AC016629.2	2.272	0.0045	FDR<0.05
AC005837.4	2.279	0.0079	FDR<0.05
AC012254.3	2.284	0.0426	FDR<0.05
LIPH	2.287	0.0021	FDR<0.05

CYP2J2	2.304	0.0340	FDR<0.05
HNF4A	2.305	0.0345	FDR<0.05
AC016745.2	2.307	0.0216	FDR<0.05
AC087292.1	2.309	0.0107	FDR<0.05
DNAJC9-AS1	2.310	0.0026	FDR<0.05
AC104758.2	2.320	0.0138	FDR<0.05
LSMEM2	2.322	0.0068	FDR<0.05
AC021491.1	2.327	0.0303	FDR<0.05
AKR1B15	2.334	0.0202	FDR<0.05
EPO	2.335	0.0130	FDR<0.05
TMEM262	2.336	0.0101	FDR<0.05
LINC02601	2.343	0.0124	FDR<0.05
AC010542.2	2.344	0.0487	FDR<0.05
AL109840.2	2.348	0.0079	FDR<0.05
AC025165.2	2.350	0.0154	FDR<0.05
LINC00334	2.351	0.0161	FDR<0.05
AL137857.1	2.354	0.0158	FDR<0.05
ESPN	2.358	0.0297	FDR<0.05
DAND5	2.360	0.0004	FDR<0.05
AL035251.1	2.366	0.0401	FDR<0.05
CCN5	2.373	0.0010	FDR<0.05
AC010503.1	2.376	0.0329	FDR<0.05
RNF39	2.377	0.0070	FDR<0.05
AC007036.1	2.381	0.0096	FDR<0.05
PTPN7	2.384	0.0022	FDR<0.05
PRR15	2.384	0.0057	FDR<0.05
ARAP1-AS1	2.396	0.0009	FDR<0.05
AC068385.1	2.416	0.0140	FDR<0.05
UGT1A7	2.423	0.0386	FDR<0.05
AC018557.1	2.426	0.0063	FDR<0.05
PTGER1	2.428	0.0016	FDR<0.05
CD27	2.430	0.0020	FDR<0.05
SCTR	2.438	0.0245	FDR<0.05
AC079742.1	2.440	0.0155	FDR<0.05
AC120498.8	2.440	0.0231	FDR<0.05
AGXT	2.443	0.0205	FDR<0.05
FXYD6-FXYD2	2.451	0.0425	FDR<0.05
Metazoa_SRP_20	2.452	0.0161	FDR<0.05
AC092171.2	2.460	0.0437	FDR<0.05
AL096855.1	2.473	0.0182	FDR<0.05
AL121845.2	2.477	0.0000	FDR<0.05
PLA2G1B	2.480	0.0007	FDR<0.05
HOXA11-AS	2.497	0.0168	FDR<0.05
Z98745.1	2.511	0.0285	FDR<0.05
AC145207.2	2.514	0.0015	FDR<0.05
AL121757.2	2.522	0.0008	FDR<0.05

AL031600.3	2.535	0.0003	FDR<0.05
ZG16B	2.537	0.0145	FDR<0.05
AC034193.1	2.550	0.0066	FDR<0.05
AC067930.1	2.552	0.0001	FDR<0.05
AL450306.1	2.557	0.0045	FDR<0.05
TREH	2.557	0.0025	FDR<0.05
AC024267.6	2.561	0.0421	FDR<0.05
AC124248.1	2.562	0.0017	FDR<0.05
BEST1	2.570	0.0000	FDR<0.05
TSGA10IP	2.575	0.0098	FDR<0.05
AC015813.4	2.577	0.0019	FDR<0.05
AL133481.1	2.577	0.0133	FDR<0.05
AC079328.2	2.581	0.0196	FDR<0.05
MT-TN	2.583	0.0011	FDR<0.05
FP236383.5	2.596	0.0104	FDR<0.05
AL391056.2	2.601	0.0011	FDR<0.05
AC099518.5	2.612	0.0034	FDR<0.05
JSRP1	2.615	0.0005	FDR<0.05
AC005253.2	2.625	0.0109	FDR<0.05
ALDH1A2	2.630	0.0169	FDR<0.05
AC008537.4	2.637	0.0020	FDR<0.05
SNX22	2.641	0.0000	FDR<0.05
TNNC1	2.654	0.0042	FDR<0.05
AC068831.4	2.656	0.0002	FDR<0.05
AQP2	2.656	0.0161	FDR<0.05
AC141586.3	2.678	0.0260	FDR<0.05
FST	2.679	0.0015	FDR<0.05
AC114498.2	2.681	0.0011	FDR<0.05
PDZK1IP1	2.684	0.0006	FDR<0.05
NR1I3	2.686	0.0082	FDR<0.05
KLK1	2.692	0.0353	FDR<0.05
MT-TS1	2.694	0.0030	FDR<0.05
AC135457.1	2.701	0.0143	FDR<0.05
AC006064.4	2.702	0.0005	FDR<0.05
FP671120.6	2.714	0.0091	FDR<0.05
AC087393.2	2.715	0.0058	FDR<0.05
AC104655.1	2.717	0.0147	FDR<0.05
AC008894.2	2.722	0.0008	FDR<0.05
AC134312.5	2.724	0.0486	FDR<0.05
AC104116.1	2.724	0.0484	FDR<0.05
AL078621.4	2.727	0.0010	FDR<0.05
LINC02273	2.729	0.0485	FDR<0.05
KIF12	2.729	0.0490	FDR<0.05
AP003352.1	2.732	0.0008	FDR<0.05
RPS6KB2-AS1	2.734	0.0159	FDR<0.05
AL158214.2	2.734	0.0073	FDR<0.05

AC016588.1	2.738	0.0314	FDR<0.05
ELANE	2.743	0.0007	FDR<0.05
NEU4	2.748	0.0179	FDR<0.05
AL033538.2	2.752	0.0164	FDR<0.05
FP236383.4	2.755	0.0094	FDR<0.05
AC124067.4	2.759	0.0248	FDR<0.05
AC125611.3	2.761	0.0045	FDR<0.05
AL499627.2	2.785	0.0174	FDR<0.05
AC018761.2	2.791	0.0015	FDR<0.05
AC010761.1	2.795	0.0024	FDR<0.05
AC006547.3	2.797	0.0294	FDR<0.05
FOXA3	2.811	0.0249	FDR<0.05
CST6	2.817	0.0079	FDR<0.05
RHBG	2.827	0.0004	FDR<0.05
LINC00336	2.853	0.0410	FDR<0.05
IGLL5	2.856	0.0315	FDR<0.05
AL359853.1	2.859	0.0420	FDR<0.05
CHI3L1	2.862	0.0045	FDR<0.05
AC092069.1	2.865	0.0027	FDR<0.05
SSTR3	2.883	0.0418	FDR<0.05
LINC02036	2.883	0.0402	FDR<0.05
NKX6-2	2.883	0.0056	FDR<0.05
PHOSPHO1	2.889	0.0146	FDR<0.05
AL590133.2	2.931	0.0013	FDR<0.05
CRYM	2.935	0.0154	FDR<0.05
AC116317.1	2.936	0.0023	FDR<0.05
AC008750.4	2.940	0.0001	FDR<0.05
AC022966.1	2.947	0.0015	FDR<0.05
AC007308.1	2.952	0.0407	FDR<0.05
RETN	2.954	0.0032	FDR<0.05
AL157871.1	2.959	0.0001	FDR<0.05
EN1	2.972	0.0139	FDR<0.05
AC091607.2	2.984	0.0032	FDR<0.05
NLRP2	2.988	0.0010	FDR<0.05
APOC4	2.992	0.0205	FDR<0.05
AC084125.1	2.994	0.0009	FDR<0.05
PVRIG	3.005	0.0258	FDR<0.05
AC093627.7	3.005	0.0072	FDR<0.05
AC008649.1	3.010	0.0143	FDR<0.05
AP003032.2	3.022	0.0171	FDR<0.05
AC020909.2	3.025	0.0121	FDR<0.05
MT-TQ	3.026	0.0008	FDR<0.05
AL391650.1	3.069	0.0011	FDR<0.05
CLEC18C	3.069	0.0361	FDR<0.05
PVALB	3.075	0.0049	FDR<0.05
TRIM72	3.091	0.0139	FDR<0.05

Metazoa_SRP_134	3.092	0.0178	FDR<0.05
HSH2D	3.096	0.0005	FDR<0.05
AC090164.2	3.099	0.0356	FDR<0.05
SLURP2	3.100	0.0044	FDR<0.05
AC091053.1	3.106	0.0003	FDR<0.05
AC020909.3	3.108	0.0098	FDR<0.05
AC109460.2	3.112	0.0011	FDR<0.05
MYOM3	3.124	0.0038	FDR<0.05
WNT7B	3.134	0.0227	FDR<0.05
AL031963.1	3.135	0.0053	FDR<0.05
AC000032.1	3.139	0.0448	FDR<0.05
LINC00844	3.140	0.0013	FDR<0.05
AC018521.2	3.164	0.0322	FDR<0.05
TMEM184A	3.173	0.0005	FDR<0.05
AC005944.1	3.176	0.0000	FDR<0.05
MUC6	3.187	0.0036	FDR<0.05
AC084024.3	3.197	0.0073	FDR<0.05
ELF3-AS1	3.199	0.0299	FDR<0.05
AC097448.1	3.225	0.0404	FDR<0.05
AL354953.1	3.231	0.0353	FDR<0.05
ATP6V0A4	3.231	0.0063	FDR<0.05
AC068888.2	3.261	0.0212	FDR<0.05
AL161452.1	3.263	0.0365	FDR<0.05
C19orf84	3.265	0.0344	FDR<0.05
AL136295.17	3.265	0.0100	FDR<0.05
HHATL	3.268	0.0000	FDR<0.05
CREB3L3	3.280	0.0022	FDR<0.05
IGFL2-AS1	3.280	0.0045	FDR<0.05
HOXD10	3.296	0.0167	FDR<0.05
AP000759.1	3.298	0.0035	FDR<0.05
AC011511.5	3.315	0.0096	FDR<0.05
AC020612.4	3.330	0.0168	FDR<0.05
AC105020.2	3.343	0.0236	FDR<0.05
AC012615.2	3.362	0.0001	FDR<0.05
C6orf47-AS1	3.368	0.0017	FDR<0.05
RN7SKP187	3.396	0.0075	FDR<0.05
ZNF32-AS1	3.400	0.0161	FDR<0.05
LEFTY2	3.410	0.0010	FDR<0.05
AC005041.3	3.411	0.0083	FDR<0.05
AC027319.1	3.413	0.0003	FDR<0.05
NKX3-2	3.439	0.0052	FDR<0.05
TRIM74	3.443	0.0011	FDR<0.05
MYL10	3.450	0.0439	FDR<0.05
AL357497.1	3.454	0.0086	FDR<0.05
AC007193.3	3.468	0.0169	FDR<0.05
BPIFB1	3.478	0.0222	FDR<0.05

PLPP2	3.484	0.0002	FDR<0.05
AC145207.3	3.502	0.0002	FDR<0.05
AC116337.3	3.504	0.0051	FDR<0.05
AP001107.2	3.521	0.0004	FDR<0.05
S100A12	3.548	0.0016	FDR<0.05
AC107081.2	3.552	0.0104	FDR<0.05
RCC2-AS1	3.603	0.0043	FDR<0.05
AC133550.2	3.623	0.0158	FDR<0.05
AC063962.1	3.626	0.0368	FDR<0.05
AC091180.3	3.669	0.0000	FDR<0.05
AC015908.7	3.717	0.0155	FDR<0.05
TRDN	3.720	0.0001	FDR<0.05
AC026954.3	3.729	0.0023	FDR<0.05
PITX1	3.733	0.0008	FDR<0.05
AL356417.1	3.737	0.0220	FDR<0.05
AC002398.2	3.752	0.0000	FDR<0.05
AC005410.2	3.760	0.0168	FDR<0.05
AL590133.1	3.771	0.0013	FDR<0.05
AC015688.6	3.787	0.0105	FDR<0.05
POU2AF1	3.801	0.0026	FDR<0.05
AC005606.1	3.816	0.0086	FDR<0.05
NKX2-3	3.826	0.0429	FDR<0.05
AC010503.2	3.826	0.0022	FDR<0.05
MT-TY	3.838	0.0019	FDR<0.05
AL117190.2	3.869	0.0001	FDR<0.05
AC026785.2	3.883	0.0088	FDR<0.05
CTRB1	3.896	0.0001	FDR<0.05
HLA-DQB1-AS1	3.903	0.0103	FDR<0.05
AC112487.1	3.908	0.0260	FDR<0.05
CATSPER1	3.946	0.0001	FDR<0.05
RN7SL134P	3.955	0.0021	FDR<0.05
BPIFB4	3.963	0.0147	FDR<0.05
AL929472.2	4.124	0.0000	FDR<0.05
AC006441.3	4.182	0.0104	FDR<0.05
LINC02065	4.197	0.0210	FDR<0.05
CTRB2	4.216	0.0010	FDR<0.05
DYNAP	4.247	0.0296	FDR<0.05
ANXA8L1	4.303	0.0060	FDR<0.05
DIO3	4.368	0.0002	FDR<0.05
AC092979.1	4.372	0.0176	FDR<0.05
MTRNR2L8	4.376	0.0000	FDR<0.05
ALX4	4.399	0.0025	FDR<0.05
AC009509.1	4.427	0.0365	FDR<0.05
MIR1302-2HG	4.458	0.0186	FDR<0.05
DPEP1	4.476	0.0000	FDR<0.05
AL049629.2	4.520	0.0012	FDR<0.05

AC133530.1	4.598	0.0021	FDR<0.05
TCF23	4.721	0.0000	FDR<0.05
PRSS22	5.224	0.0077	FDR<0.05
LINC01096	5.420	0.0104	FDR<0.05
IGFN1	5.779	0.0000	FDR<0.05
SMIM22	6.401	0.0003	FDR<0.05
