# Supplementary figures and images for: PPARG dysregulation as a potential molecular target in adrenal Cushing's syndrome
Source: Front Endocrinol (Lausanne). 2023 Nov 30;14:1265794. doi: 10.3389/fendo.2023.1265794 (PMC10720662; doi:10.3389/fendo.2023.1265794)

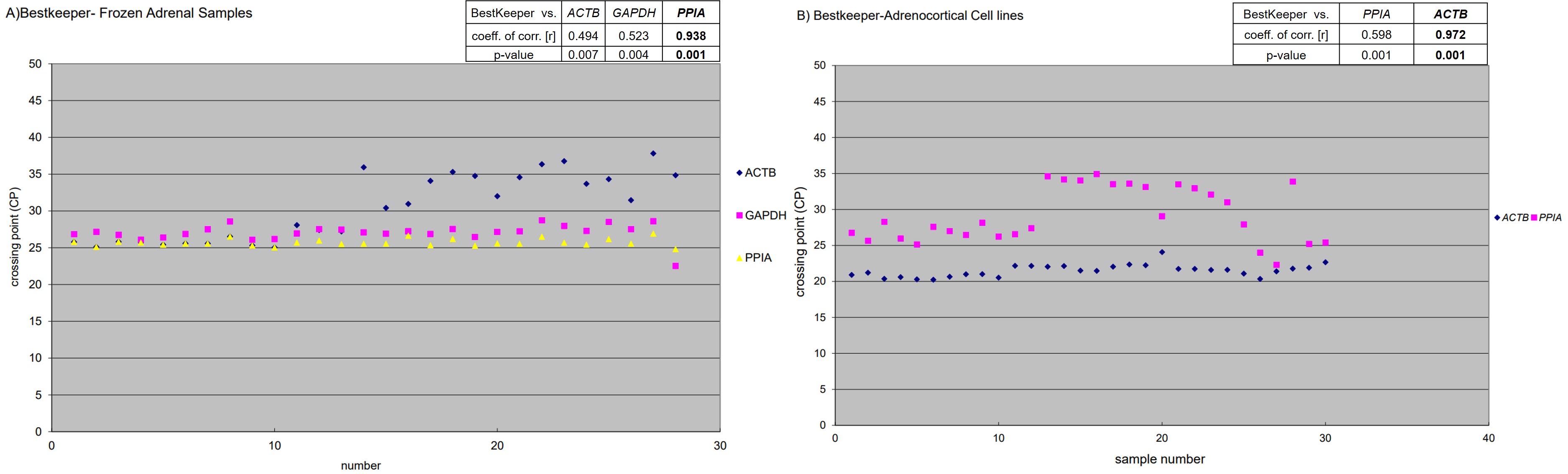

Supplement: Supplementary file 1 [file DataSheet_1.zip › supplementary 2023/FigS1 2023.jpg]

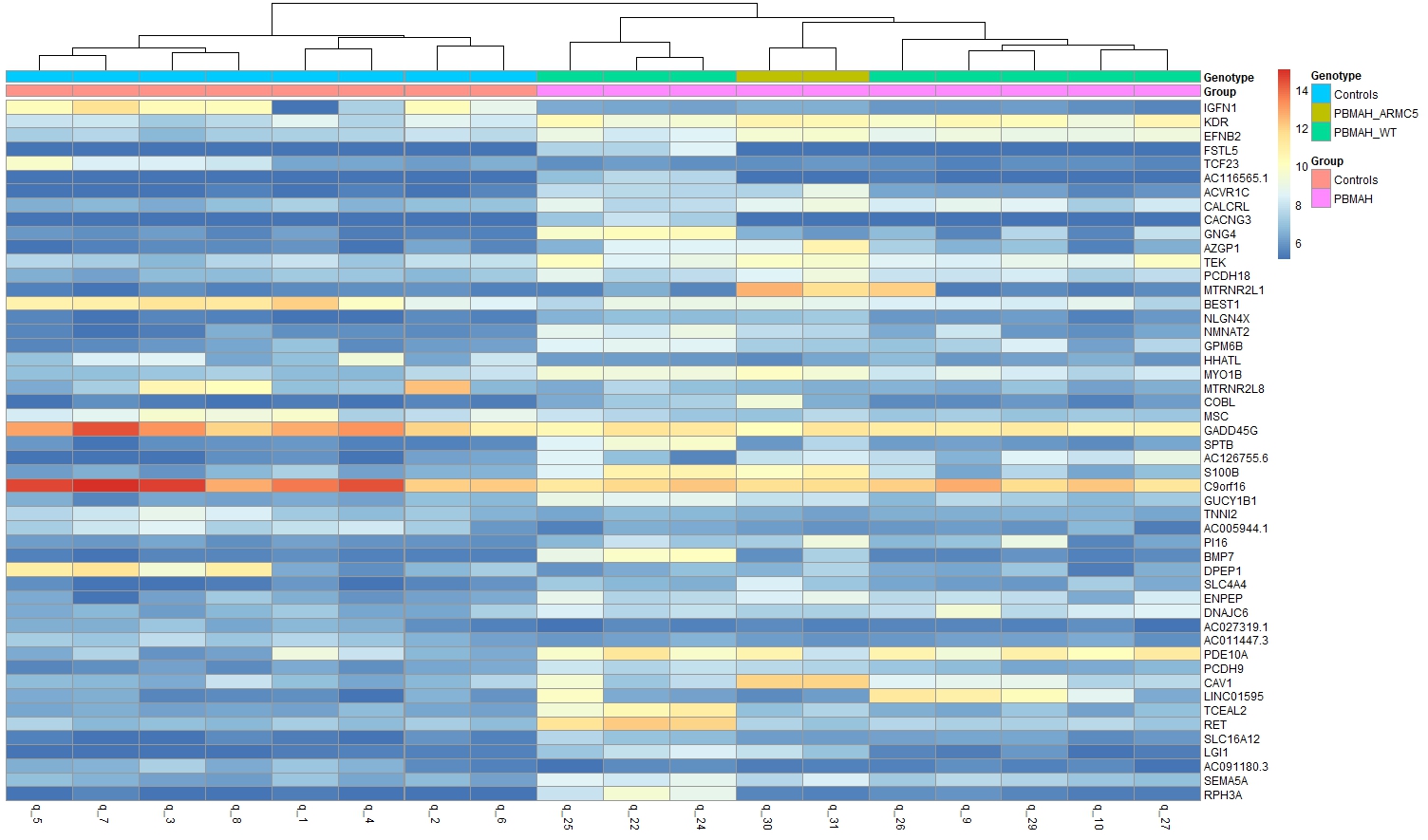

Supplement: Supplementary file 1 [file DataSheet_1.zip › supplementary 2023/FigS2 2023.jpeg]

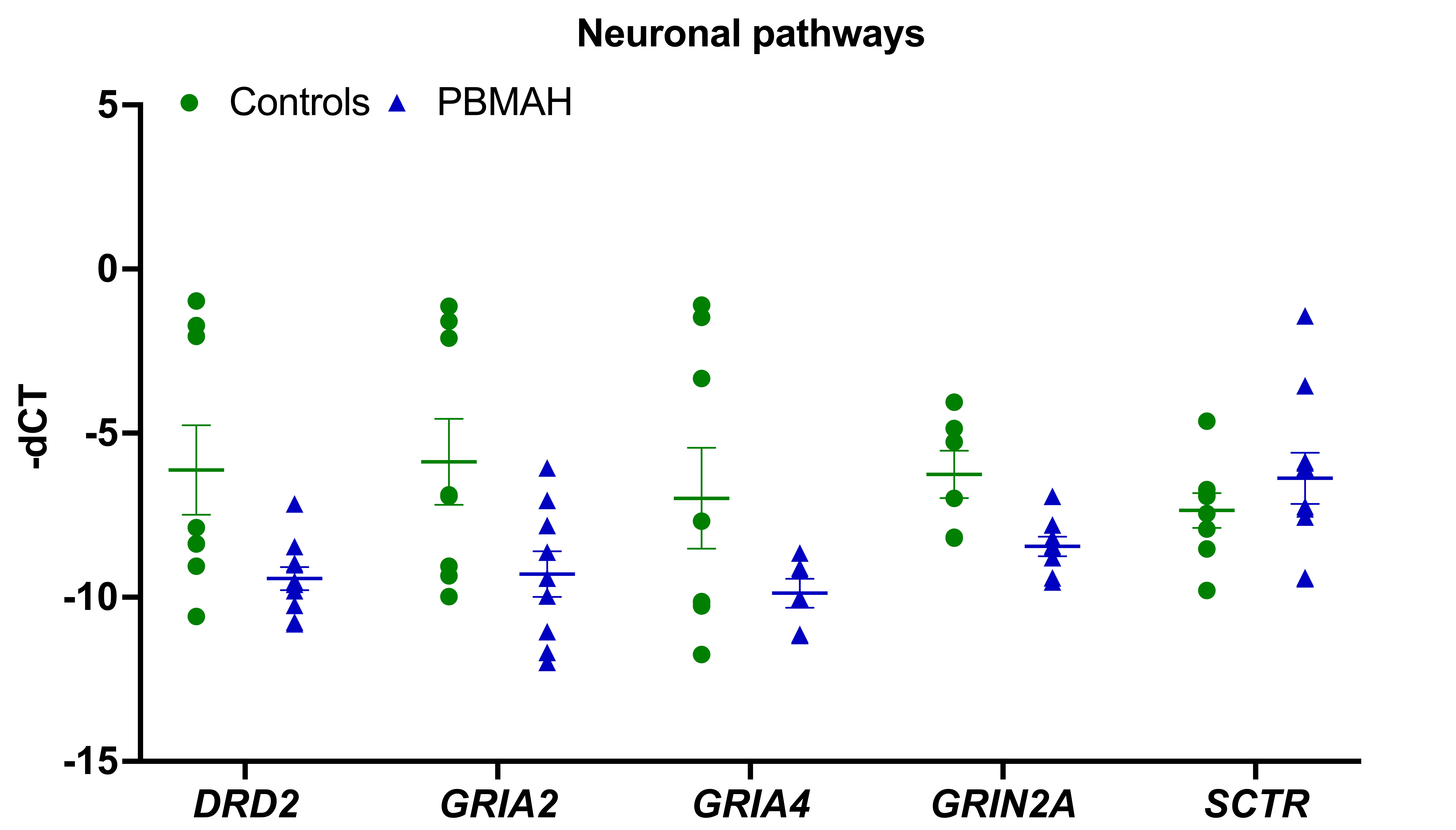

Supplement: Supplementary file 1 [file DataSheet_1.zip › supplementary 2023/FigS3 2023.jpg]

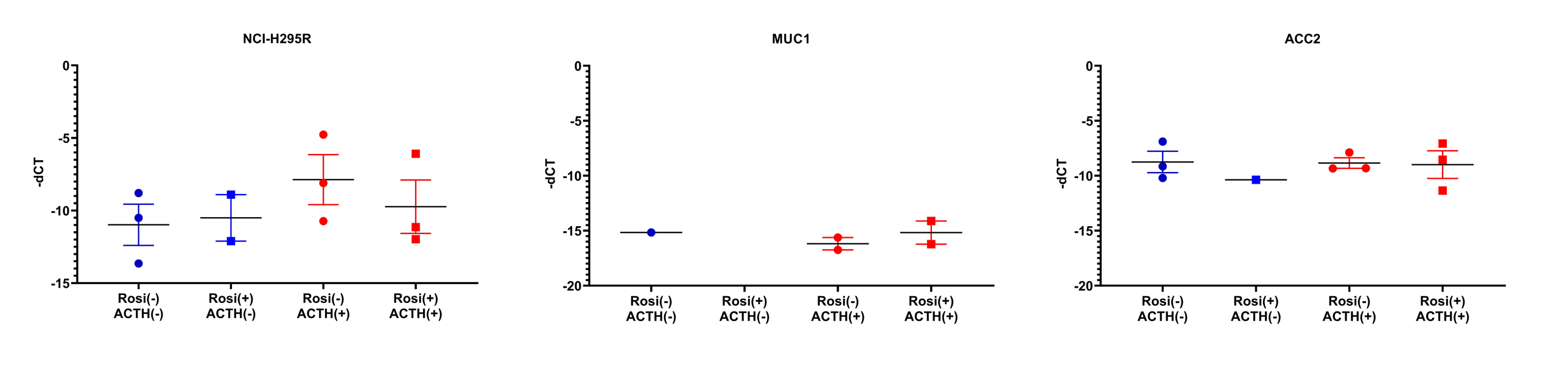

Supplement: Supplementary file 1 [file DataSheet_1.zip › supplementary 2023/FigS6 2023.jpg]
